# Use of RFID technology to characterize feeder visitations and contact network of hummingbirds in urban habitats

**DOI:** 10.1371/journal.pone.0208057

**Published:** 2018-12-12

**Authors:** Ruta R. Bandivadekar, Pranav S. Pandit, Rahel Sollmann, Michael J. Thomas, Scott M. Logan, Jennifer C. Brown, A. Peter Klimley, Lisa A. Tell

**Affiliations:** 1 Department of Medicine and Epidemiology, School of Veterinary Medicine, University of California Davis, Davis, California, United States of America; 2 EpiCenter for Disease Dynamics One Health Institute, School of Veterinary Medicine, University of California Davis, Davis, California, United States of America; 3 Department of Wildlife Fish and Conservation Biology, University of California Davis, Davis, California, United States of America; 4 Wild Wings Ecology, Sherman Oaks, California, United States of America; 5 United States Fish and Wildlife Service, Sacramento, California, United States of America; University of South Carolina, UNITED STATES

## Abstract

Despite the popular use of hummingbird feeders, there are limited studies evaluating the effects of congregation, sharing food resources and increased contact when hummingbirds visit feeders in urban landscapes. To evaluate behavioral interactions occurring at feeders, we tagged 230 individuals of two species, Anna’s and Allen’s Hummingbirds, with passive integrated transponder tags and recorded their visits with RFID transceivers at feeders. For detecting the presence of tagged birds, we developed an RFID equipped feeding station using a commercially available antenna and RFID transceiver. Data recorded included the number of feeder visits, time spent at the feeder, simultaneous feeder visitation by different individuals, and identifying which feeders were most commonly visited by tagged birds. For the study period (September 2016 to March 2018), 118,017 detections were recorded at seven feeding stations located at three California sites. The rate of tagged birds returning to RFID equipped feeders at least once was 61.3% (141/230 birds). Females stayed at feeders longer than males per visit. We identified primary, secondary and tertiary feeders at Sites 2 and 3, according to the frequency of visitation to them, with a mean percentage of 86.9% (SD±19.13) visits to a primary feeder for each tagged hummingbird. During spring and summer, hummingbirds visited feeders most often in morning and evening hours. Feeder visits by males overlapped in time with other males more frequently than other females. The analysis of the contact network at the feeders did not distinguish any significant differences between age or sex. Although most hummingbirds visited the feeders during the daytime, our system recorded night feeder visitations (n = 7 hummingbirds) at one site. This efficient use of RFID technology to characterize feeder visitations and contact networks of hummingbirds in urban habitats could be used in the future to elucidate behaviors, population dynamics and community structure of hummingbirds visiting feeders.

## Introduction

Effects of artificial resource provisioning on avian ecology, such as survival, range extension and species conservation, are poorly understood [1]. In general, the community structure of common avian species found in gardens has been known to be affected positively by artificial feeding in urban areas [2, 3]. Along with large-scale supplemental seed feeding of garden birds, use of hummingbird feeders is widespread in the Americas with many types of hummingbird feeders sold in the retail market. Not only do homeowners use feeders to attract hummingbirds, but feeders are often present at tourist attractions [4]. Despite the common use of hummingbird feeders, the benefits and detriments of supplemental sugar water feeding have been rarely studied.

Hummingbirds have the highest known metabolic rates among vertebrates and therefore require a high energy diet [5]. On one hand feeder visitations might (1) reduce foraging time, (2) sustain healthy body condition, and (3) increase reproductive success given this readily available high energy food source while on the other hand, it could (1) inflate of the carrying capacity of an urban habitat, (2) cause feeding on a less diverse food source, (3) alter pathogen transmission within population, and (4) promote unnatural congregation of different species in large numbers thus aiding in aggression between individuals [6].

To understand the effects of artificial resource provisioning on the ecology and community structure of hummingbirds, it is critical to quantify feeder usage such as the number of visits by an individual bird, identify feeders most commonly visited, and characterize behavioral patterns, such as resource guarding, associated with the feeder. Furthermore, temporal and seasonal variations in feeder usage are crucial for linking artificial resource provisioning with things related to species conservation such as disease transmission and phenology.

Traditionally, identification of individual hummingbirds has been achieved using leg bands. This method has been widely used for tracking hummingbird movements and the local presence of an individual bird [7]. However, this method does not enable investigators to quantify hummingbird behaviors such as feeder visits or bird interactions at feeders. An alternative to banding is the use of passive integrated transponders (PIT) [8–10]. This automated detection of individuals at a feeder is advantageous because it results in minimal bird disturbance and provides accurate records of the arrival and departure times of each PIT tagged individual at a feeder. Radio frequency identification (RFID) technology equipped transceivers have been used to describe the energetics [9], cognitive abilities [10] and presence/absence [11] of hummingbirds at feeders. Use of this technology eliminates the repetitive capture of individuals, and the feeder visitation behavior of birds is not altered due to trapping [11]. However, in previous hummingbird studies using RFID technology, only a single bird visiting a feeder could be recorded at any given time thus rendering this method less useful for detecting interactive behaviors, such as simultaneous visitation to a feeder by multiple birds. To date, no studies have quantified the degree of hummingbird interactions at feeders. Establishing contact network structures within hummingbird populations is pivotal for behavior studies studying interactions or for being able to model changes in transmission dynamics of infections. Many properties related to contact network have been known to be associated with disease transmission in passerine birds [1, 6, 12–17] and contact networks have also been widely used in wildlife for modeling pathogen transmission, and to identify super spreaders within the communities [18]. Risk of infections such as salmonellosis [19, 20] or pox [21–23] has been hypothesized to be associated with feeder usage in birds. Pox viral infections have been documented in hummingbirds [22], so being able to elucidate whether hummingbird feeders play a role in the transmission of this disease would be beneficial.

The objectives of this study were two-fold. The first aim of the study was to assess the effectiveness of the feeder system equipped with RFID technology to record visits by multiple PIT tagged hummingbirds. The second study objective was to extract feeder visitation metrics from the data and evaluate the contact network of these PIT tagged hummingbirds at the feeders.

## Materials and methods

### Study sites

The study was conducted at three locations in the state of California, two sites in Yolo County and one in Los Angeles County. Site 1 (38° 32' 51'' N, 121° 51' 23'' W), where a feeding station was deployed, was at private landowner’s property in Winters, CA at an elevation of 41 meters above sea level. At this site, hummingbirds were fed using commercial feeders for past 16 years and it consisted of a private garden surrounded by cultivated land. The habitat was riparian as Putah Creek passed along the site’s north side. The second site (138° 31' 49'' N, 121° 45' 42'' W) was on the University of California, Davis (UCD) campus 16.8 meters above sea level, also in proximity of a riparian habitat. No hummingbird feeders had been deployed here prior to the study. This site was surrounded by buildings on the north and west sides and the arboretum along the east and south sides. Sites 1 and 2 were separated by 6.4 kilometers. Site 3 (34° 5' 13'' N, 118° 24' 51'' W) was on a private landowner’s property at an elevation of 247.2 meters above sea level and feeders had been deployed here for decades. The site was located on a hill surrounded by residential homes on one side and an undeveloped canyon consisting of native vegetation on the other.

### Subjects

Anna’s (n = 167; *Calypte anna*) and Allen’s Hummingbirds (n = 63, *Selasphorus sasin*) were tagged with passive integrated transponders (PIT) between September 2016 to March 2018 and October 2017 to March 2018, respectively (Table 1). Individuals were captured using a drop net feeder trap, identified, aged, and sexed as previously described [24]. Later in the calendar year, when both hatch year and after-hatch year birds had molted, some birds could not be aged due to a lack of bill corrugations and presence of adult plumage. The age of these birds was designated as “unknown”. All birds were visually examined and those with no visible signs of compromised health and /or disease condition(s) were tagged. Body weight was also a factor taken into consideration to assess the health of the bird. Durations of monitoring for both hummingbird species at all three sites are listed in Table 2.

**Table 1:** Demographic composition of hummingbirds (n = 230) tagged with passive integrated transponders at Sites 1 and 2 in Northern California and Site 3 in Southern California.

Species	Female			Male			Total
	After- Hatch Year (AHY)	Hatch Year (HY)	Unknown	After- Hatch Year (AHY)	Hatch Year (HY)	Unknown	
**Allen’s Hummingbird** *Selasphorus sasin*	6	0	8	26	2	21	**63**
**Anna’s Hummingbird** *Calypte anna*	27	23	18	39	40	20	**167**
**Total**	**33**	**23**	**26**	**65**	**42**	**41**	**230**

**Table 2:** Number of hummingbirds PIT tagged at Sites 1 and 2 in Northern California and Site 3 in Southern California. Open feeders are defined as feeders that did not have radiofrequency identification equipment. Feeding stations are defined as those equipped with radiofrequency identification equipment to detect passive integrated transponders.

Site	Data Collection Time Frame	Allen’s Hummingbirds *Selasphorus sasin*	Anna’s Hummingbirds *Calypte anna*	Total	Number of open feeders	Number of feeding stations
**Site 1**	September 2016—March 2018	0	53	**53**	3	1
**Site 2**	May 2017—March 2018	0	36	**36**	0	3
**Site 3**	October 2017—March 2018	63	78	**141**	12	3
	**Total**	**63**	**167**	**230**	15	7

All procedures related to this study were approved by the UC Davis Institutional Animal Care and Use Committee (Protocol: 20355), United States Geological Survey Bird Banding Laboratory (Permit: 23947), United States Fish and Wildlife (Permit: MB55944B-2), and California Department of Fish and Wildlife (Permit: SC-013066).

#### PIT tagging of hummingbirds

**Fig 1:**
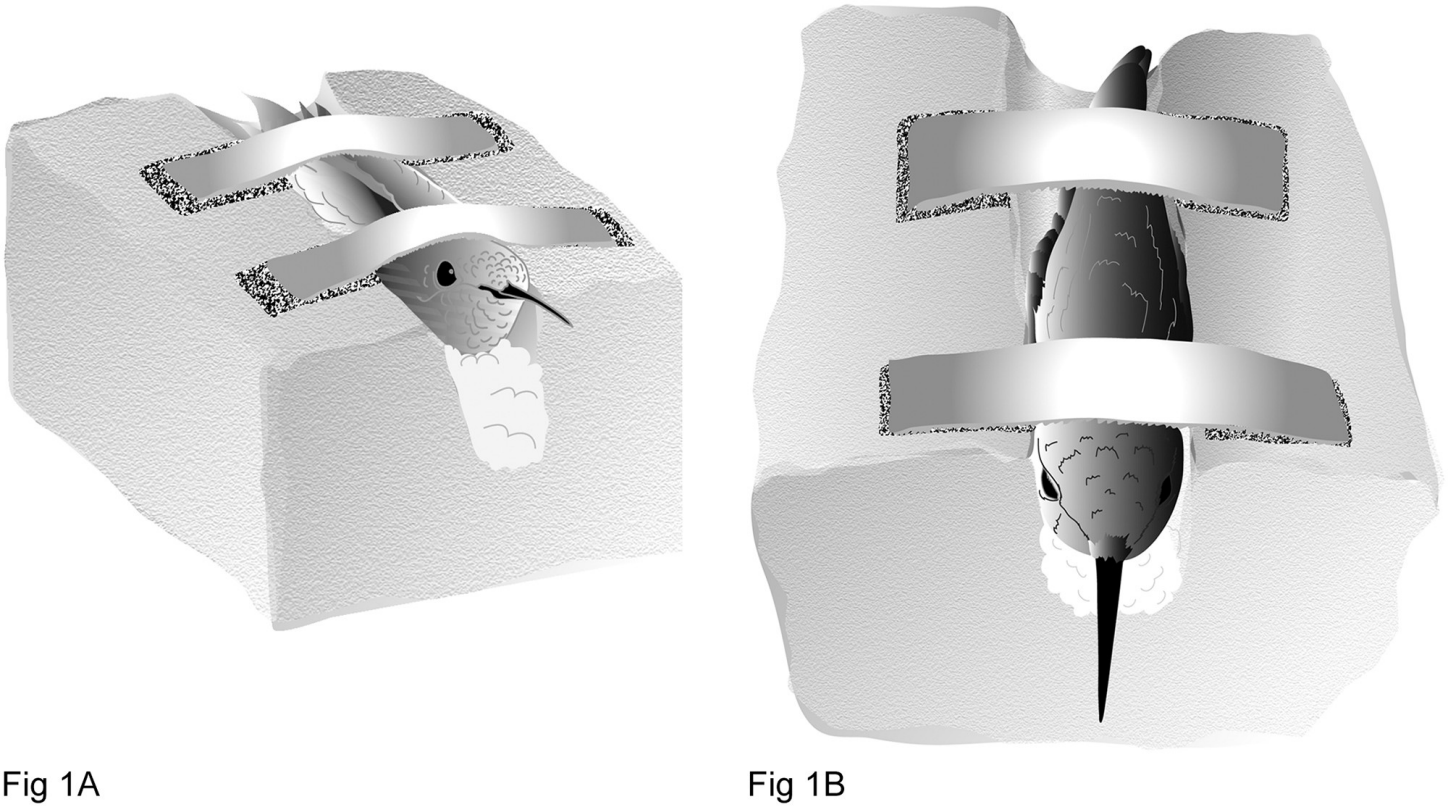
Side (A) and top (B) view of polyethylene foam (1.7 pound per cubic foot) block used to restrain and secure hummingbirds for subcutaneous insertion of the passive integrated transponder in the dorsal lumbar region of the bird. Note that the bird holding reservoir is beveled so that the top portion is wider than the bottom section. The polyethylne foam is firm enough to secure the bird, but flexible enough not to restrict respiratory movement. Two re-closable fastener strips are used to hold the bird in place and a bird is placed in the block so that the wings are secure.

Hummingbirds were tagged with 8 mm long passive integrated transponders (PIT) of 134.2 kHz frequency (mini HPT8, Biomark, Boise, ID). PITs were either glued (n = 7) or subcutaneously implanted (n = 223) in the dorsal region as previously described [25]. A polyethylene foam (1.7 pound per cubic foot) block with a beveled holding reservoir (Fig 1) was used to secure the bird using re-closable fastener strips (3M) as previously described [25]. The PIT did not exceed 3% of the bird’s body mass, which is consistent with the United States Geological Survey Bird Banding Laboratory (BBL) policy. For initial 7 birds, the tags were secured on the skin and feathers of the dorsum using eyelash glue [10]. For the remaining birds, PITs were inserted subcutaneously using 16-gauge needles (N165 injector needle, Biomark,) and a syringe with a long stylet (MK 165 implanter, Biomark). PITs were placed in the lumen just inside the bevel opening at the most distal end of the injector needles and sterilized using a gas hydrogen peroxide system (AMSCO V-Pro 1 Low-Temperature Sterilization System). Prior to injecting the PIT under the skin, the feathers on the dorsal aspect of the bird were parted using a moistened cotton tip applicator with 0.13% benzalkonium chloride and 2.5% lidocaine hydrochloride (pain relieving cleansing spray; Bactine). The feathers were separated so that the underlying skin was exposed, and an injection site could be identified. The dorsal ridge of the thoracic and lumbar vertebrae was used as an anatomical landmark to guide tag placement to either side of the bird’s body. Prior to injecting with the RFID loaded needle, the stylet was injected through the distal end of the needle just until it met resistance due to the presence of the tag. This helped ensure that the stylet would enter the lumen of the needle without resistance prior to starting the injection process. The insertion site for the needle was in the caudal half of the body and special attention was paid so that the needle bevel was always face up. When inserting the needle through the skin, care was also taken to ensure that the needle tip remained above the plane of the lumbar muscles. This prevented inadvertent nicking of the underlying muscle fascia. The needle was injected just until the bevel portion was under the skin and then the stylet was pushed forward to inject the tag under the skin. For 26 birds, the insertion site was closed using surgical glue (Surgical adhesive, Vet one). For 197 birds, the insertion site was closed using a single interrupted suture with either 4–0 polydioxanone (PDS) II (#98SUT6-22, Ethicon) or 5–0 polyglycolic acid (#S-G518R13-U, Ethicon). The PIT tag number was read and recorded prior to and after the placement using a commercial handheld reader (GPR PLUS, Biomark). Leg bands were placed on all tagged hummingbirds so that loss of a PIT could be ascertained upon bird recapture.

### Monitoring technology

For detecting the presence of tagged birds, we developed a RFID technology incorporated feeding station that included a hummingbird feeder, a netted structure surrounding the feeder, and a commercially available antenna and RFID transceiver. antenna feeding station. Each antenna was connected to a RFID transceiver (HPR PLUS.05V1, Biomark). All feeding stations were situated such that no perches (natural or artificial) were present within the range of the antenna of the feeding stations.

**Fig 2:**
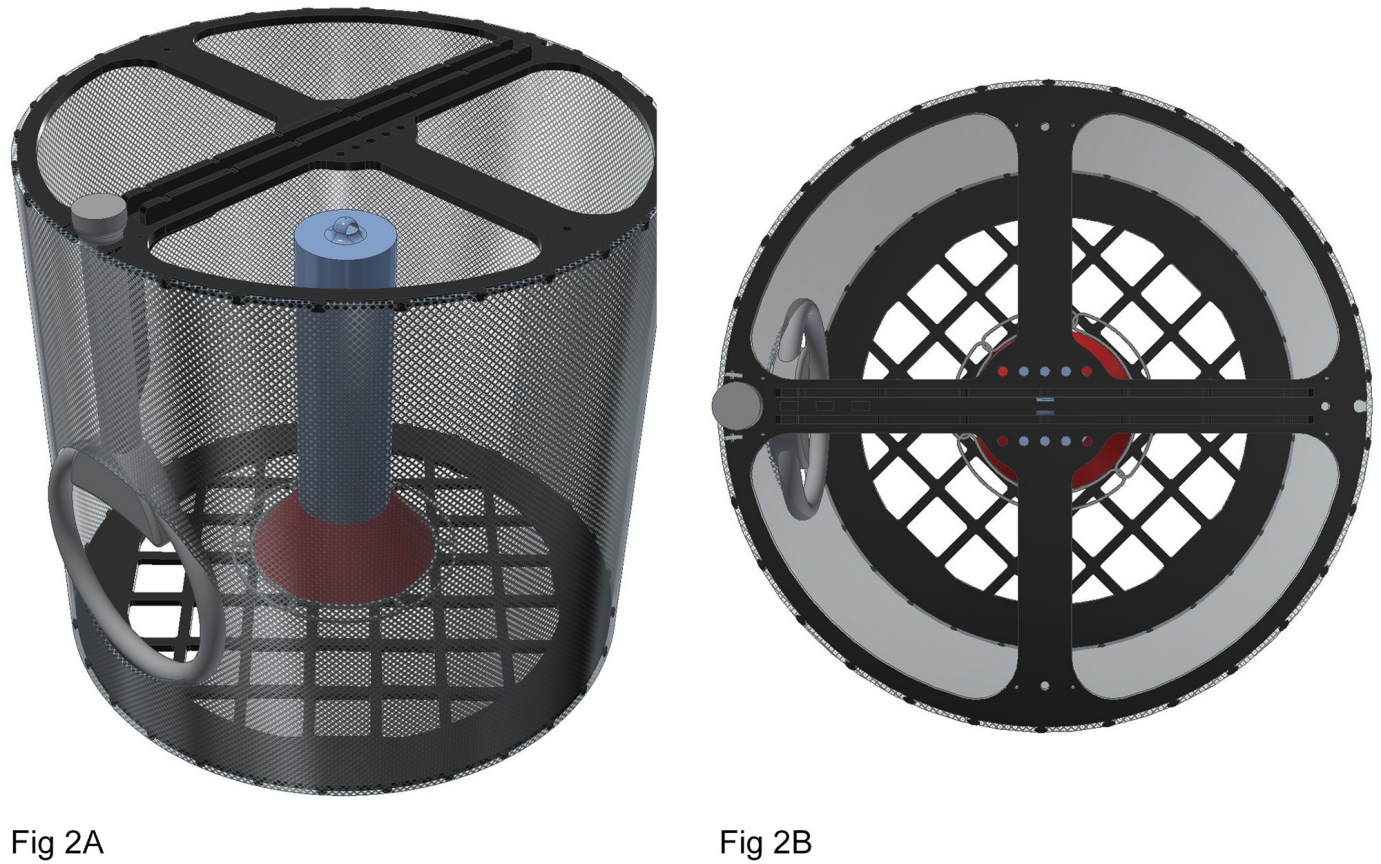
Computer-aided design drawings of (A) side and (B) top views of the single antenna feeding station using radiofrequency identification technology. The antenna was secured to the side of the feeder system using commercial cable ties that secured the antenna to the supporting perpendicular structure that originated from the top of the unit that extended down (A). The crossbars had drilled mounting holes, for cord string to be fed through, allowing suspension of the entire unit (B). The top portion of the unit is devoid of netting and serves as a secondary exit route for the birds.

Two types of RFID feeding stations were evaluated in this study: a single antenna feeding station and a double antenna feeding station. The single antenna feeding station (Fig 2) consisted of two 45.72 cm diameter circular discs separated by 50.8 cm of nylon netting (3/4^th^ inch, Bird-X) and was fit with a commercial feeder (Hummingbird Feeder 209B, 887 ml Perky Pet, 30 oz). The top circular disc was devoid of netting, allowing for an escape route for the birds, and had two crossbars that provided structural support for suspension. A commercial rope was fed through the crossbars’ drilled with holes for mounting the unit. The antenna was attached to the side of the feeder system using cable ties that secured the antenna to the supporting perpendicular structure that originated from the top circular disc. A hole was cut in the net to accommodate the loop antenna (15.2 cm diameter). The birds’ port of entry was through the loop antenna. The feeder was suspended so that it was at the same level as the opening of the antenna, thus aiding in attracting birds while providing a directional sense of entry. The single antenna feeding station was elevated 1.2 m above ground using bent irrigation tubing that served as a frame for suspension. A weight was attached to the feeding station to minimize its movement. The RFID transceiver was housed in a covered plastic bucket and powered from an AC outlet.

**Fig 3:**
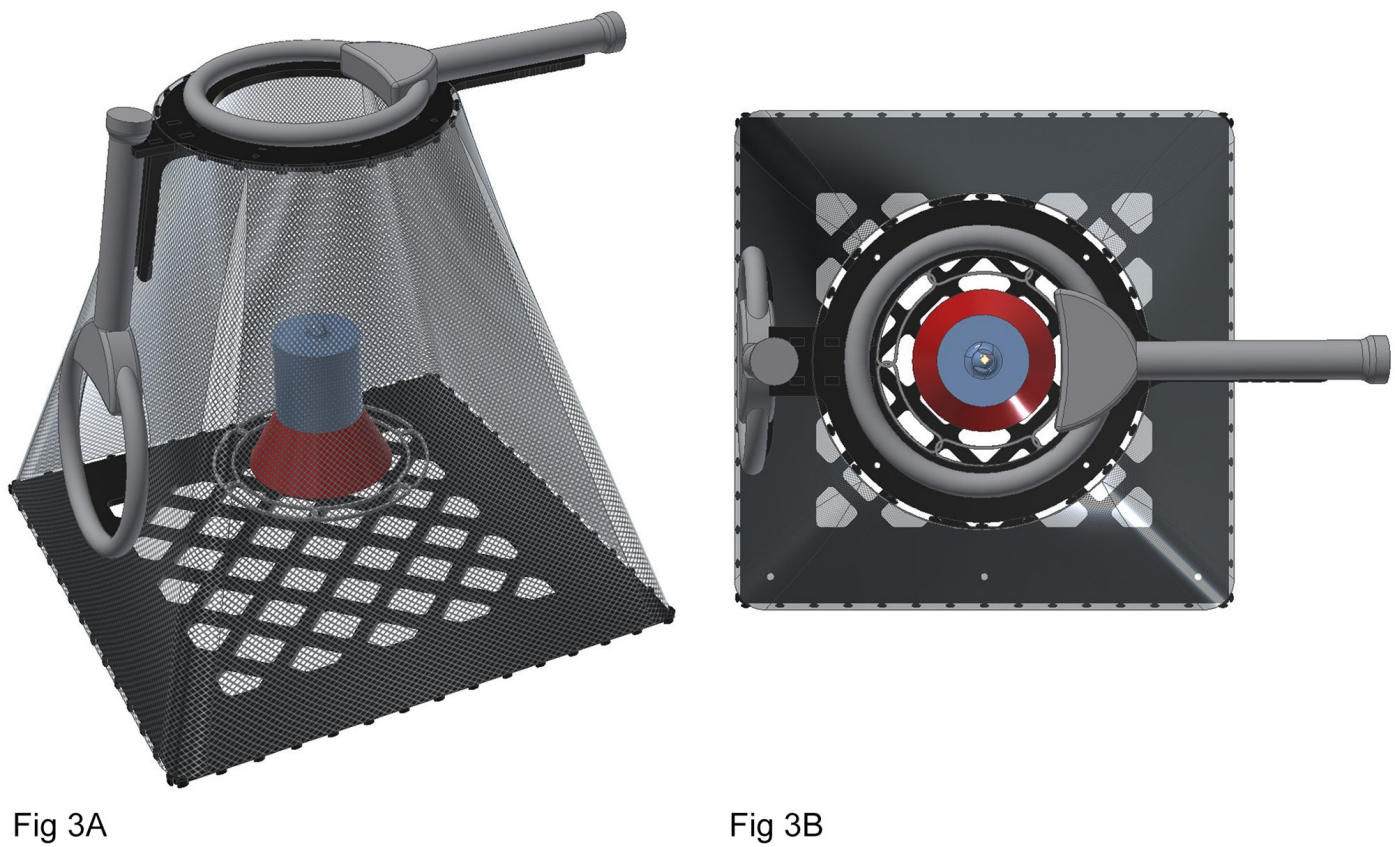
Computer-aided design drawings of (A) side and (B) top views of the double antenna feeding station using radiofrequency identification technology. The feeder was positioned in the unit by mounting it on ¾ inch diameter metal pole that was driven into the soil bed. The top portion of the unit is devoid of netting and serves as a secondary exit route for the birds.

The double antenna feeding station had a cubical shape (Fig 3). It was ensured that hummingbirds were able to exit the unit and were not getting trapped in a corner or a region with the folded netting. The double antenna feeding station consisted of a smaller square plate (20.32 cm diagonal) separated from the lower square plate (50.8 cm diagonal) using nylon net. Owing to the reduced inner space, a smaller commercial feeder (993091–001, 473.18 ml; First nature, 12 oz), with the same number of artificial flowers (6) as the feeder used in the single antenna feeding station, was placed in the double antenna feeding station and the feeder itself was suspended from the bottom of the feeder’s saucer with a metal pole. Separate RFID transceivers were attached to each antenna on this feeding station and they independently detected the presence of PIT tagged hummingbirds.

Prior to connecting antennas to feeding stations, they were tested to ensure uniformity and to confirm the expected PIT detection range (25–30 cm from the center of the antenna in each direction). The antennas were then attached to the feeding stations so that, in theory, the distance from the center of the antenna to the farthest feeder port could be read.

The RFID transceivers were programmed with the following settings for recording hummingbird detections. For Site 1, the transceiver was initially set to read the same tag twice after an interval of 2 minutes, but nine months into the study the detection rate was changed to 10 secs between detections from the same bird. For Sites 2 and 3; the transceivers were set to re-read a tag at 10 second intervals as long as the bird was continuously in the range of the antenna.

At Site 2, the feeding stations were placed in a triangular configuration approximately 1–2 meters apart in the northwest corner of the UC Davis Arboretum Teaching Nursery. Feeding stations were placed on the side of the house that faced the undeveloped canyon and were approximately 3–4 meters away from the house at Site 3. The feeders were in three distinct areas of the yard approximately 10 meters apart.

### Data analysis

Visualization of data trends, and data analysis were performed using the Python and R programming languages. The code used for data management, statistical analyses and to generate figures is accessible at the following repository: https://zenodo.org/record/1467481#.W85-9UtKiUk. When available, data for both Anna’s and Allen’s Hummingbirds were analyzed unless specified otherwise. The primary assessment of system was done by two ways 1) by comparing the detections by side and top antenna of the double antenna feeding station 2) by estimating the survivability of tagged birds in the study. For all the statistical analysis, a *p*-value less than 0.05 was considered as significant.

#### Comparison of data output from double antenna feeding station

For the double antenna RFID feeding unit, to determine if the top antenna recorded similar numbers of tag reads, and individual bird tags compared the side antenna, tag data from the two antennas were compared with each other. Comparisons were made with respect to the number of visits detected as well as the number of unique birds detected.

#### Survival analysis

We used capture-recapture models to determine whether the single antenna feeding station could serve as a survey method for estimating hummingbird population parameters. Capture-recapture models enable the use of repeated detection/non-detection data to estimate population size while accounting for imperfect detection of individuals [26, 27]. Specifically, we applied a Cormack-Jolly-Seber (CJS) [28, 29] model to monthly files of tag detections recorded between September 2016 until September 2017 for hummingbirds tagged at Site 1 in September 2016. This enabled us to estimate survival rates and evaluate whether these differed among males and females, or hatch year (HY) and after-hatch year (AHY) individuals. In the CJS model, individuals are marked, in this case, tagged with PITs, and subsequently recaptured, in this case, detected by the system. Only marked animals are considered in the analysis. Non-observation of an individual could be the result of lack of detection due to birds avoiding feeding stations or the result of death or emigration from the study area. The CJS model cannot separate emigration from mortality and thus estimates apparent survival, i.e., the product of survival and site fidelity. Apparent survival probability refers to the time interval between subsequent occasions. To convert RFID data to the CJS format, we divided the duration of the study into 13 month-long samples and noted whether an individual was recorded at least once during each sample. Consequently, estimates of survival probability refer to monthly survival. This approach drastically reduces the information from the continuous acquisition of PIT detections, but because we implemented the survival analysis as a proof of concept, we used this standard analytical approach rather than exploring a continuous-time model. We identified the effect of multiple covariates on survival probability and detection probability, including the season (Winter: Dec, Jan, Feb; Other: Mar—Nov), sex and age (AHY or HY, from tagging data). We compared models using the Akaike Information Criterion adjusted for small sample size [30] in two steps [28]. First, we tested the effect of individual covariates on detection while including all three covariates on survival. Second, based on the top detection model (i.e., the detection model with the lowest AICc), we explored individual covariates on survival. For completeness, we included a model without covariates on detection or survival (full null model) in both model selection steps. We considered models with ΔAIC<2 (i.e., within 2 units of AICc from the top model) to have strong support. We implemented models and model selection in R version 3.4.3 [31] using RMark 2.2.4 [32], an R package that links to Program MARK [33].

#### Feeder visitation

**Fig 4:**
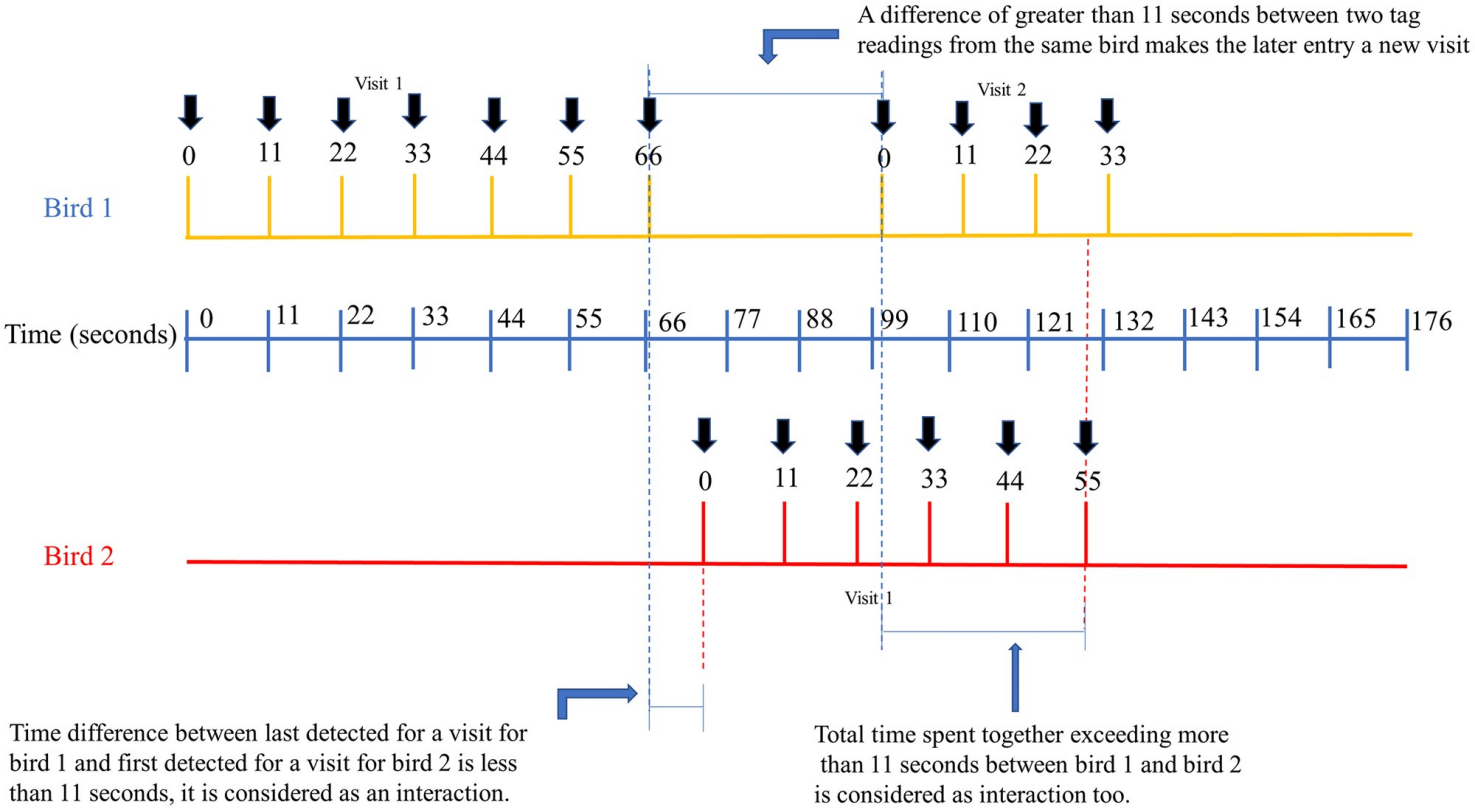
Figure showing passive integrated transponder tag detections of two hummingbirds on time axes and illustrating the estimation of visit duration and hummingbird interactions.

The difference between two consecutive detections equal to 11 seconds was considered to be the same “visit” for an individual bird. The duration of the visit was defined as the difference between the earliest tag detection and the latest detection (Fig 4). The mean visit duration, total time spent at the feeder and the proportion of time spent at the feeder by hummingbirds were compared between age and sex groups using Kruskal-Wallis Test. Proportion of time spent at the feeder was defined as the ratio between total time spent and the duration of observation for the bird (difference between first and last detected in the system). For all birds visiting the feeder, we also calculated the time for tagged birds to make their first feeder visit after tagging and number of days birds visited feeders. For Sites 2 and 3, we identified primary, secondary and tertiary feeders based on bird feeder visitation numbers for individual birds for all RFID equipped feeders and differentiated primary, secondary and tertiary feeders for individual birds at the site using Kruskal-Wallis Test.

#### Diel variation by Anna’s Hummingbirds

The diel variation (variation in the daily pattern) of feeder visitation by Anna’s Hummingbirds was evaluated using the Rayleigh’s test to test the uniformity in the distribution of mean hourly visits by birds [34]. Diel variation was also evaluated by sex for Anna’s Hummingbirds.

#### Contact network

Interactions between two tagged individuals were identified by detecting an overlap of visitation times by two individual hummingbirds on the same feeding station. We defined an interaction to be short when the time difference between the end of the earlier visit and start of the successive visit was less than 11 seconds. A long interaction was defined as when the co-mingling time of any two PIT- tagged hummingbirds was more than 11 seconds (Fig 4). A static network of birds interacting with each other was constructed to identify community structure within populations. Birds involved in interactions were considered nodes and edges between two nodes indicated the presence of an interaction. The weight of the edge was proportional to the interaction time. The network was drawn using force atlas-2, a force-directed drawing algorithm [35]. The degree of a node was defined as the number connections it has with other birds in the network and betweenness centrality was defined as the number of times the bird acts as the shortest path between two other birds. We used generalized linear models based on node level permutations to identify the effects of gender and age of individuals on their centrality measures (degree and betweenness centrality) in the network with 10,000 permutations.

## Results

### Animal subjects

During the tagging process, when the birds were restrained in the foam holder for tag placement, they exhibited normal respiratory patterns. During the process of subcutaneous tag injection, no morbidity or mortality was observed for either hummingbird species. Immediately following PIT tag placement, all birds were able to fly. On rare occasion (< 10/223) hemorrhage occurred when the underlying muscle fascia was nicked by the injection needle, but the hemorrhage was controlled by applying localized digital pressure. Injection sites for birds that were observed upon recapture (n = 53 birds) were unremarkable. For the entire study duration, none of the birds were found trapped within the single or double antenna RFID tagging stations.

### Detections of PIT tagged hummingbirds

A total of 118,017 tag detections were recorded by the RFID transceivers and represented 65,476 visits by 141 PIT tagged hummingbirds to seven feeding stations from September 2016 to March 2018 (Table 3). 61.3% of the PIT tagged hummingbirds (75.9% Anna’s Hummingbirds and 24.1% Allen’s Hummingbirds) were detected at least once by the readers. 35.5% of the PIT tagged females and 64.5% of the PIT tagged males were detected at least once independent of the species. Independent of hummingbird species, 30.5% HY and 43.4% AHY and 26.2% of unknown aged hummingbirds were detected at least once by our readers feeder systems. The median for the time span between tagging and the first detection by the RFID transceiver was 5 days (mean = 31.4 days, SD = 58 days, range 0–444 days). Tagged birds were detected on an average 24.6 days (SD = 44.3, range 1–289 days) with the median of 4 days throughout their observation period (S1 Table). The median observation period (difference between the PIT tagging date and the date the bird was last detected by the RFID transceiver) for tagged hummingbirds was 33 days (mean = 88.8 days, SD = 110 days, range 1–554 days).

**Table 3:** Demographic composition of tagged hummingbirds visiting feeding stations at Sites 1 and 2 in Northern California and Site 3 in Southern California from September 2016 to March 2018. Allen’s Hummingbirds were tagged only at Site 3 in Southern California.

Species	Female			Male			Total
	After- Hatch Year (AHY)	Hatch Year (HY)	Unknown	After- Hatch Year (AHY)	Hatch Year (HY)	Unknown	
**Allen’s Hummingbird** *Selasphorus sasin*	**3**	**0**	**6**	**12**	**1**	**12**	**34**
**Anna’s Hummingbird** *Calypte anna*	**18**	**17**	**6**	**28**	**25**	**13**	**107**
**Total**	**21**	**17**	**12**	**40**	**26**	**25**	**141**

Based on recaptures (n = 43), since birds were both banded and PIT tagged, it was determined that none of the recaptured birds lost their PIT tags regardless of the time that they were recaptured after tag placement (PIT tag injection sites were observed as early as one day after tag placement and as late as day 355 following tag placement) or the number of recaptures (subsequent recaptures ranged from one [n = 41 hummingbirds] which were up to four recaptures per bird [n = 12 hummingbirds]).

#### Comparison of data output from double antenna feeding station

**Fig 5:**
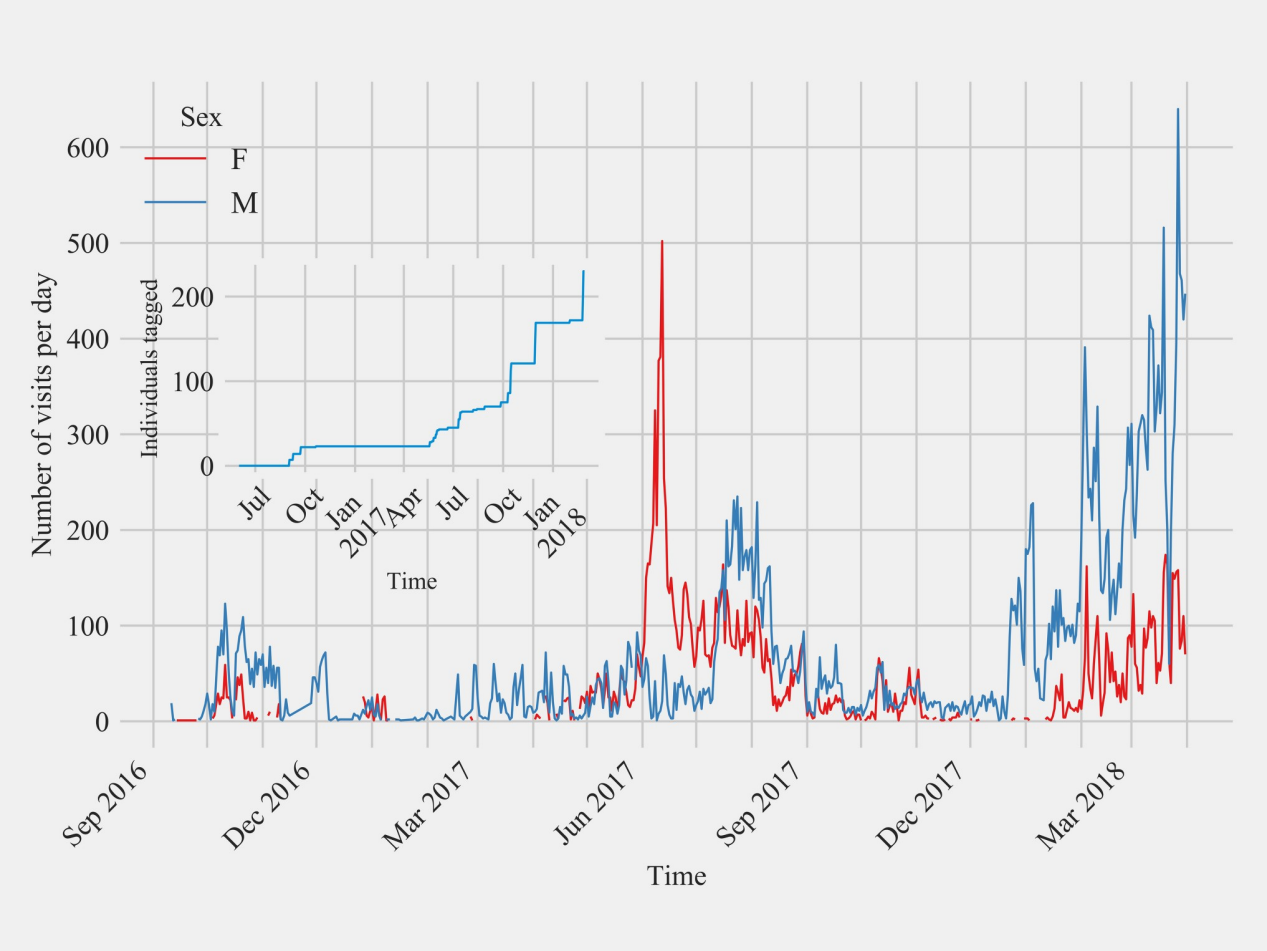
Comparison of the daily detections from the two different antennas at the double antenna feeding station transceiver at Site 2 over time. The top antenna for this feeding station was deployed in May 2017 and discontinued in January 2018 for data collection. Overall, the total number of visits detected by the side antenna exceeded the number of visits for the top antenna but not enough to warrant the addition of a second antenna. Both antennas detected the presence of the same number of individual birds.

Comparisons of the number of tag detections by side antenna and the top antenna of the double antenna feeding station at Site 2 exhibited similar temporal trend (Fig 5). Both antennas detected the same twenty PIT tagged birds, but the side antenna detected slightly more feeder visits (7,825 visits) compared with the top antenna (7,620 visits) during the period from May 2017 to January 2018. Therefore, for the double antenna feeding station we only considered tag detections from the side antenna for all analyses.

#### Survivability

The Cormack-Jolly-Seber (CR) model was applied to detections at Site 1 between September 2016 and September 2017. This included records for 33 PIT tagged individuals. Of these, 22 were female (7 AHY, 15 HY) and 11 were male (4 AHY, 7 HY) birds. Fourteen tagged birds were detected at least once by the single antenna feeding station; the mean number of detections for each bird at the single antenna feeding station was 3.9. The CR model indicated that only age was a statistically significant predictor of detection probability (Table 4). Based on a detection model including age, the model with the lowest AICc score was the null survival model (i.e., no covariates on survival; Table 4). For both model selection steps, the full null model performed much worse (ΔAICc>10) than the top model. Estimated monthly survival probability under the top model was 0.76 (SE 0.05); detection probability was estimated at 0.91 (SE 0.09) for AHY and at 0.39 (SE 0.08) for HY.

**Table 4:** Cormack-Jolly-Seber model selection results for Anna’s Hummingbirds, stratified by age, sex and season for the tagged population at Site 1 between September 2016–2017.

Model		Npar	AICc	Δ AICc	Weight
**Detection Models**	Phi (Δ~ Season + Sex + adult) p (~adult)	6	163.13	0.00	0.95
	Phi(~1) p(~1)	2	169.33	6.20	0.04
	Phi (Δ~ Season + Sex + adult) p (~1)	5	174.01	10.88	0.00
	Phi (Δ~ Season + Sex + adult) p (~sex)	6	175.51	12.39	0.00
	Phi (Δ~ Season + Sex + adult) p (~season)	6	175.55	12.42	0.00
**Survival Models**	Phi (~1) p (~adult)	3	159.29	0.00	0.37
	Phi (~adult) p (~adult)	4	159.92	0.63	0.27
	Phi (~Season) p (~adult)	4	160.31	1.02	0.22
	Phi (~sex) p (~adult)	4	161.22	1.93	0.14

### Feeder visitations

**Fig 6:**
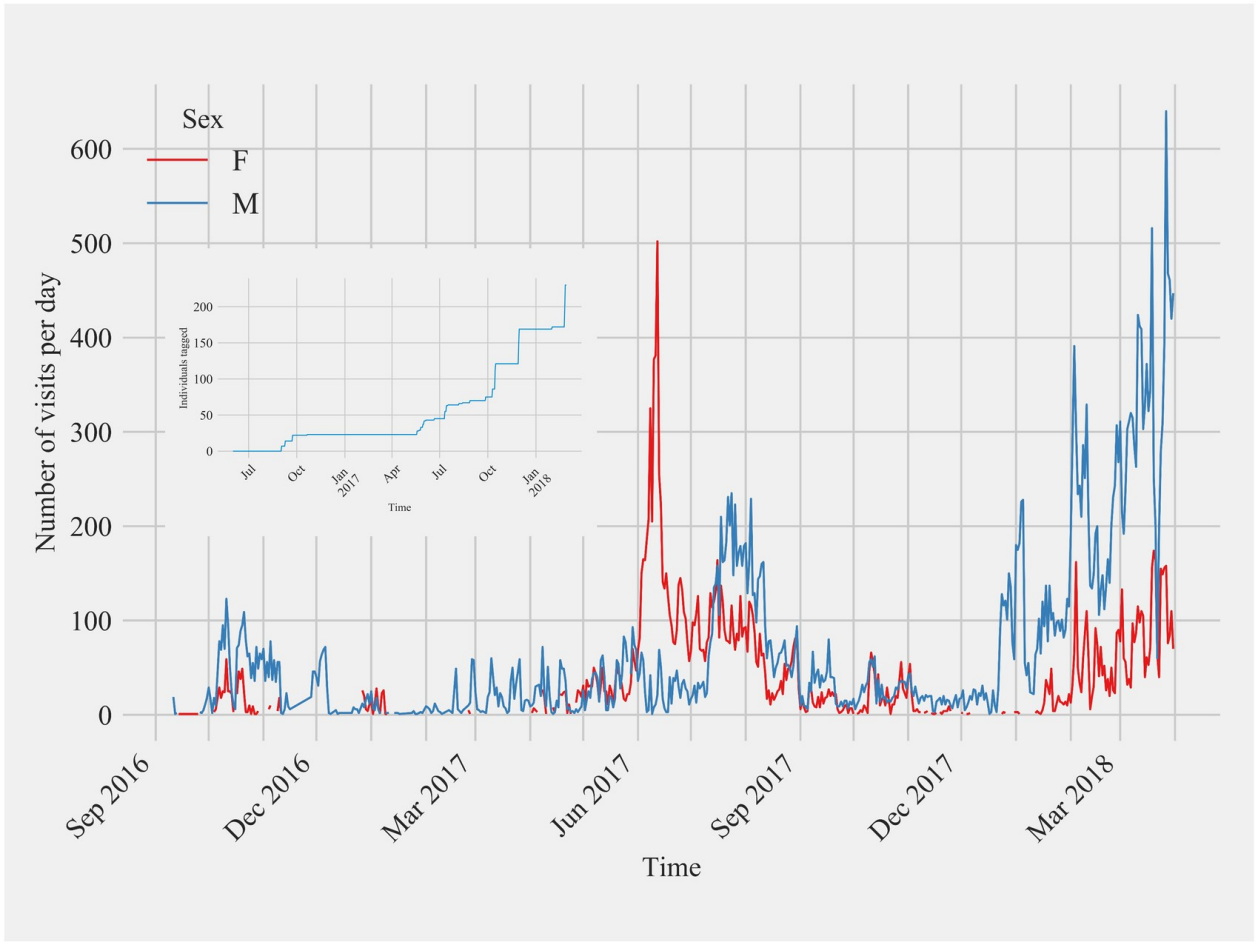
Daily visitations of passive integrated transponder tagged male and female Anna’s Hummingbirds at the feeding stations at Sites 1 and 2 in Northern California and Site 3 in Southern California from September 2016 to March 2018. Inset: The cumulative tagging effort throughout the study.

There were 65,476 visits by 141 PIT tagged hummingbirds to seven feeding stations from the three sites. The mean duration of visits was 7.3 seconds (SD = 17.4, range = 11–615 seconds) with most visits being transient visits with duration less than ten seconds (median = 0.0 seconds, as least count of the system is 10 seconds). Hummingbirds spent a mean time of 00:50:53 hrs. (SD = 02:20:55 hrs., range = 00:00:11–16:38:42 hrs.) at feeders throughout the study (S1 Table). Seventy-one out of 141 detected birds spent time at feeders only with transient visits (visits with duration less than ten seconds), which are detected as visits with duration equals to zero by the system. Hence, the distribution of total time spend by birds was skewed with a median equal to zero seconds. Out of 65,476 hummingbird visits to the feeding stations, 48,713 visits were transient visits that were less than 10 seconds in duration. The remaining 16,763 visits were of longer duration encompassing a total of 5 days 05 hours 1 minute and 30 seconds of the observation period. The average duration of longer visits between females (mean = 25.11 seconds, SD = 11.96, n = 25) and males (mean = 23.43 seconds, SD = 11.27, n = 45) were statistically similar (Kruskal-Wallis, *p* < 0.61). On average, birds were present 0.07% (SD = 0.0013) time at the feeding station out of total observed duration. No statistical difference (*p* = 0.46) was detected between males and females for the proportion of time spent at the feeder. Limited local movement of PIT tagged hummingbirds visiting feeding stations at different sites was observed with only four Anna’s Hummingbirds (3 HY males, 1 HY female) tagged at Site 2 visiting Site 1 during the study. Of the three males, one male Anna’s Hummingbird had 8.9% of its visits at Site 1 with rest of the visits (91.1%) at Site 2, whereas the other two Anna’s Hummingbird males spent only 0.6% and 0.2% of their visits at Site 1. The female hatch year Anna’s Hummingbird that traveled to Site 1 from Site 2 spent 1.0% of her visits at Site 1. Feeder visits over time for the two sexes of Anna’s Hummingbird at all three sites are shown in Fig 6. There was a peak in the visits by female Anna’s Hummingbirds a month earlier than males in 2017, but in general, females were most frequently detected during July and males during August. Male Anna’s Hummingbirds were detected during January 2018 with few detections of females. Both were detected during early spring 2018.

### Primary—Tertiary hummingbird feeders for individual birds

**Fig 7:**
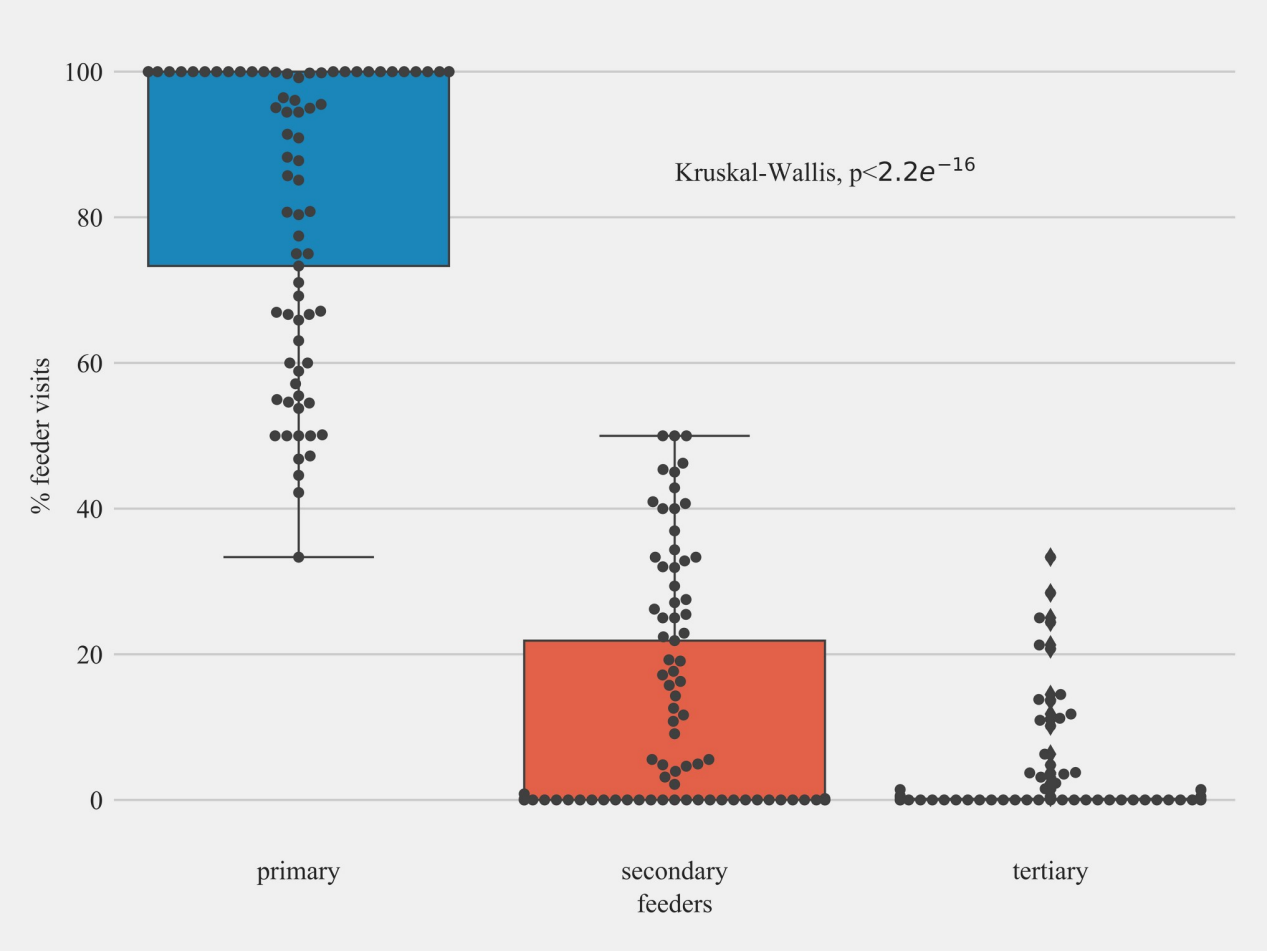
Feeder visit percentages for individual Anna’s and Allen’s Hummingbirds for primary, secondary, and tertiary feeders at Site 2 in Northern California. Solid circles show the data points.

Data from Sites 2 and 3 showed that individual hummingbirds had a pattern where they visited one feeder more often compared to other feeders. Individual hummingbirds had a different primary, secondary and tertiary feeders. On an average of 86.7% (SD = 19.2;) visits were to a primary feeder (most visited feeder for individual hummingbirds), followed by 10.8% (SD = 15.6;) visits to a secondary feeder, and 2.5% (SD = 6.4;) visits to a tertiary feeder (Fig 7). At Site 2, ‘A8’ was the most visited feeder (primary feeder for 14 birds), followed by ‘A4’ (primary feeder for 10 birds), and ‘A5’ was the primary feeder for 9 birds. Similarly, at Site 3, ‘B2’ was the primary feeder for 32 birds followed by ‘A9’ (primary feeder for 26 birds), and ‘B1’ was the primary feeder for 11 birds.

### Diel rhythmicity of feeder visitations by Anna’s Hummingbirds

**Fig 8:**
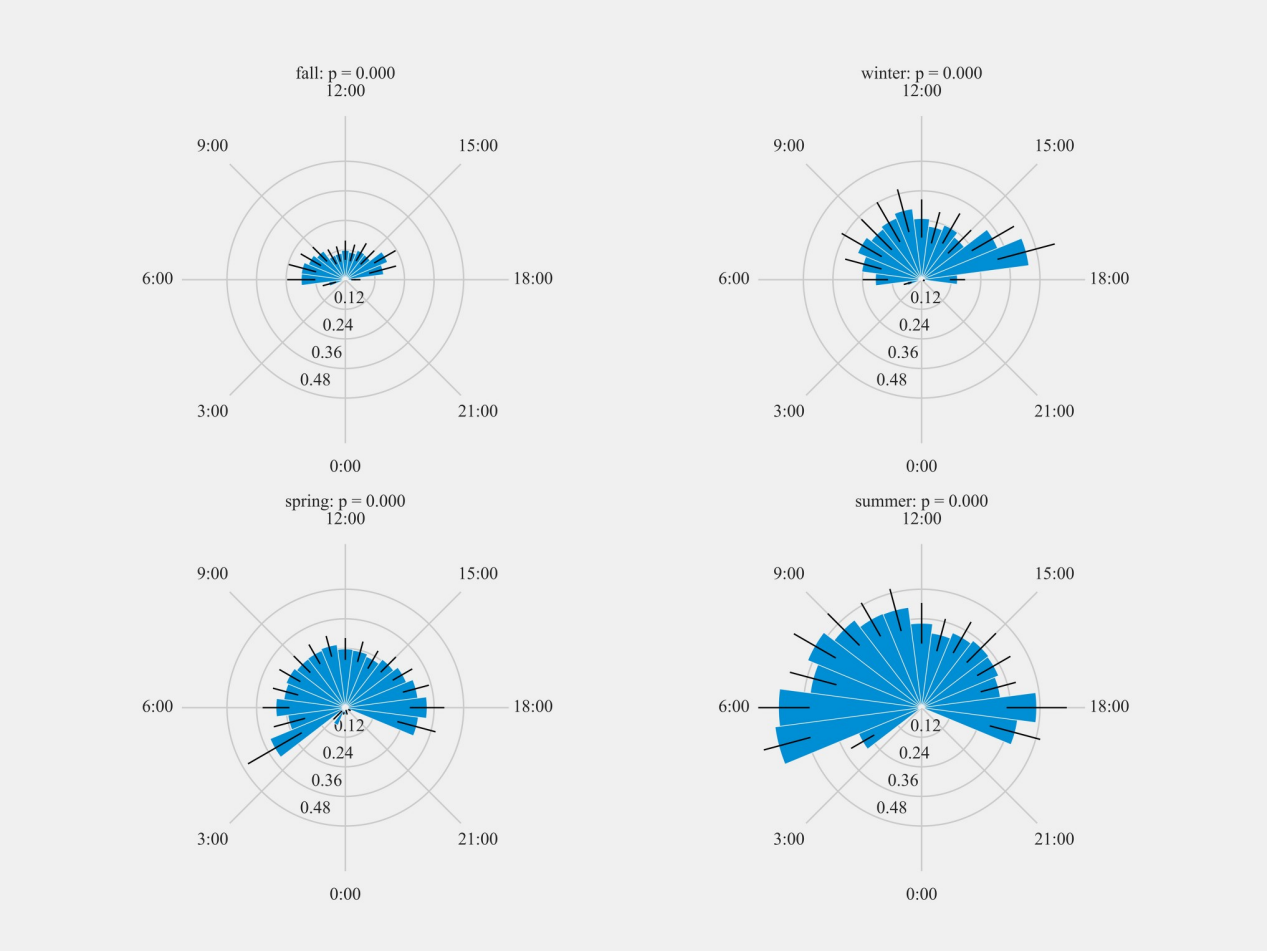
Diel plots of mean hourly Anna’s Hummingbird visits to the feeding stations at three sites at northern and southern California from September 2016 to March 2018. The bar denotes the mean hourly visits by hummingbirds and black lines show the standard error. All distributions were statistically non-uniform (probability values reported by season).

The distribution of mean hourly visits to the feeding station for Anna’s Hummingbirds was non-uniform throughout the day for all seasons (*p* < 0.005; Fig 8). The daily distribution of feeder visits by Anna’s Hummingbirds differed between seasons. Within the fall season, a considerable decrease in the hourly visits by the Anna’s Hummingbirds was observed. Conversely, a higher number of hourly visits were recorded during the months of summer. During the fall season, hummingbirds were active on an average from 05:40 until 18:10 hrs. with maximum activity between 15:00 and 16:00 hrs. In winter, birds were active from 00:02 to 23:52 hrs. with peak activity observed between 16:00 and 17:00 hrs. During spring, the earliest visit was at 00:10 hrs. and the latest one at 23:53 hrs. with the maximum activity between 17.00 to 18.00 hrs. Hummingbirds were active from 04:17 until 19:54 hrs. during the summer with the highest activity between 05:00 and 06:00 hrs. followed by another peak of activity between 17.00 to 18.00 hrs.

**Fig 9:**
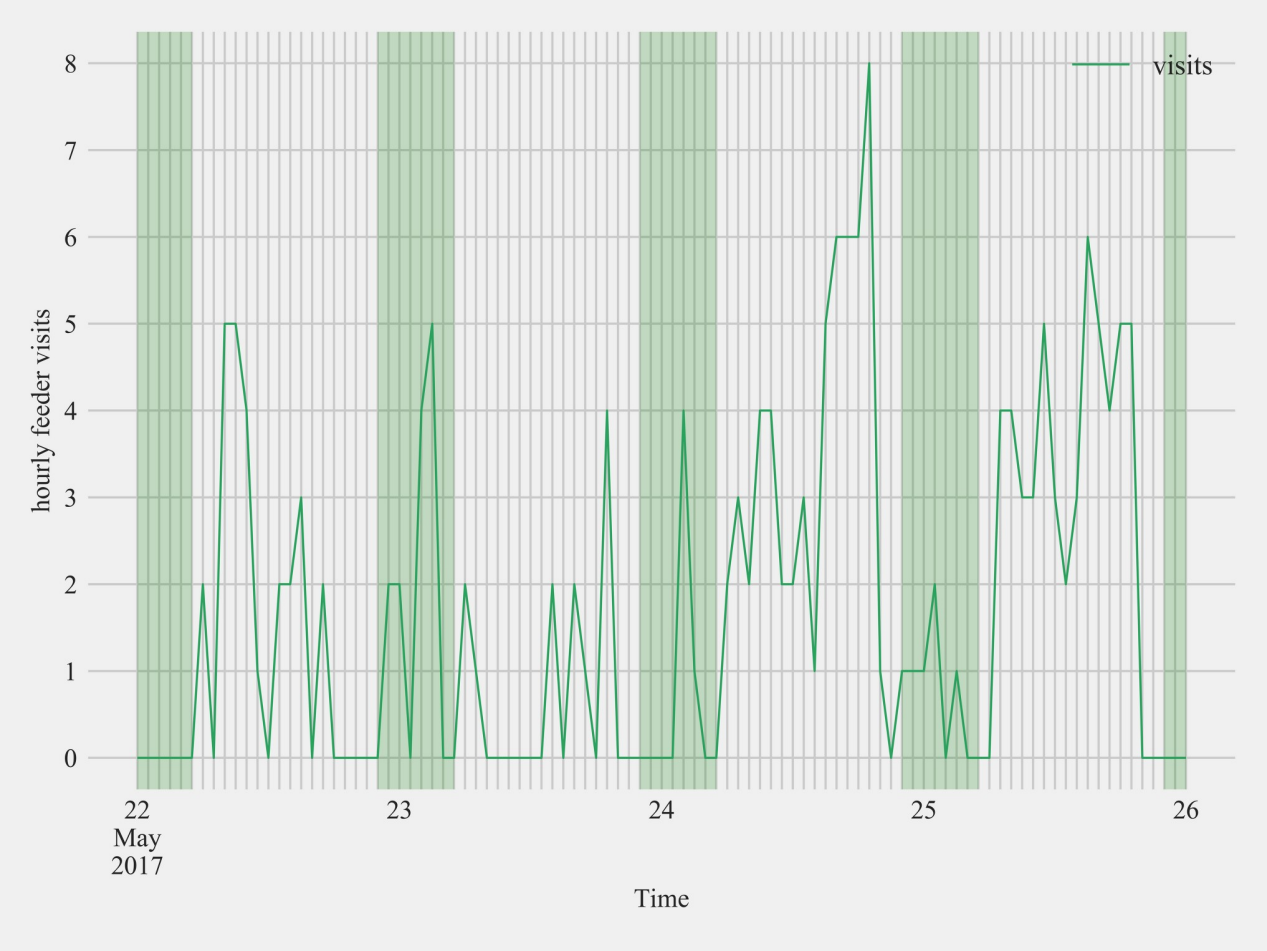
Feeder visit activity of an after-hatch year male Anna’s hummingbird with a subcutaneously placed passive integrated transponder at Site 2 between May 22nd and May 26th 2017. This bird was detected at the feeder after sunset, evident in the peaks of hourly visits during the night (green shaded sections of plot).

Seven birds (5 males and 2 females) were active between 22:00 and 04:00 hrs. during the study period. Out of the birds active at night, all five males were AHY and both the females were HY (S1 Fig). All the night activity was at Site 2 during winter and spring seasons. One AHY male was active on two feeding stations during the night hours. Another AHY male was active only on unit 2 (Fig 9) while remaining all other 5 birds were active only at unit 1 during the night hours. When the diel rhythmicity was explored separately for female and male Anna’s Hummingbirds, similar patterns were observed (S2 and S3 Figs, respectively).

### Contact network

The data showed that multiple hummingbirds visited the feeders at the same time. Their interactions were defined as the near simultaneous detection of two birds at the feeder as shown in Fig 4. Throughout the study period, total of 54.6% hummingbirds participated in 1,635 interactions (Table 5). Of the total number of birds interacting, 32.5% were females and 62.3% were males of both Anna’s and Allen’s Hummingbirds. There was no statistically significant difference in the proportion of males and females participating in interactions (females 50.0% and males = 52.7%, Fisher’s exact *p* = 0.86). A total of 11.7% of the interacting birds were Allen’s Hummingbirds, while the rest 83.1% were Anna’s Hummingbirds. Out of the 1,635 observed interactions, 1,608 were transient interactions with interaction times less than eleven seconds. The remaining 27 interactions lasted longer than eleven seconds. Higher numbers of interactions were recorded between two males (1,020 interactions), followed by interactions between a male and a female (491 interactions). The smallest number of interactions was recorded between two females (124 interactions).

**Table 5:** The age classes of hummingbirds (n = 77 birds) interacting at feeders at Site 1 and Site 2 in Northern California and Site 3 in Southern between September 2016 –March 2018.

Species	Female			Male			Total
	After- Hatch Year	Hatch Year	Unknown	After- Hatch Year	Hatch Year	Unknown	
**Allen’s Hummingbird** *Selasphorus sasin*	0	0	1	3	1	4	9
**Anna’s Hummingbird** *Calypte anna*	10	14	0	18	17	5	64
**Total**	10	14	1	21	18	9	77

**Fig 10:**
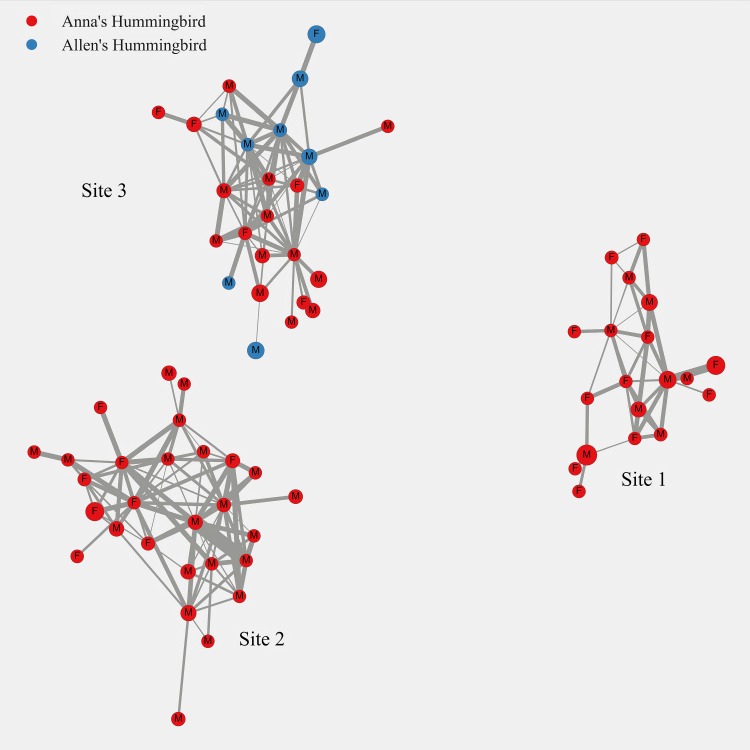
Contact network of tagged Anna’s (pink nodes) and Allen’s hummingbirds (green nodes). The sex of the hummingbirds indicated by M for males and F for females. The size of the node is proportional to the degree of the interaction. Edges represent time spent together at the feeding station and the width of the edge width is proportional to the time spent together.

**Fig 11:**
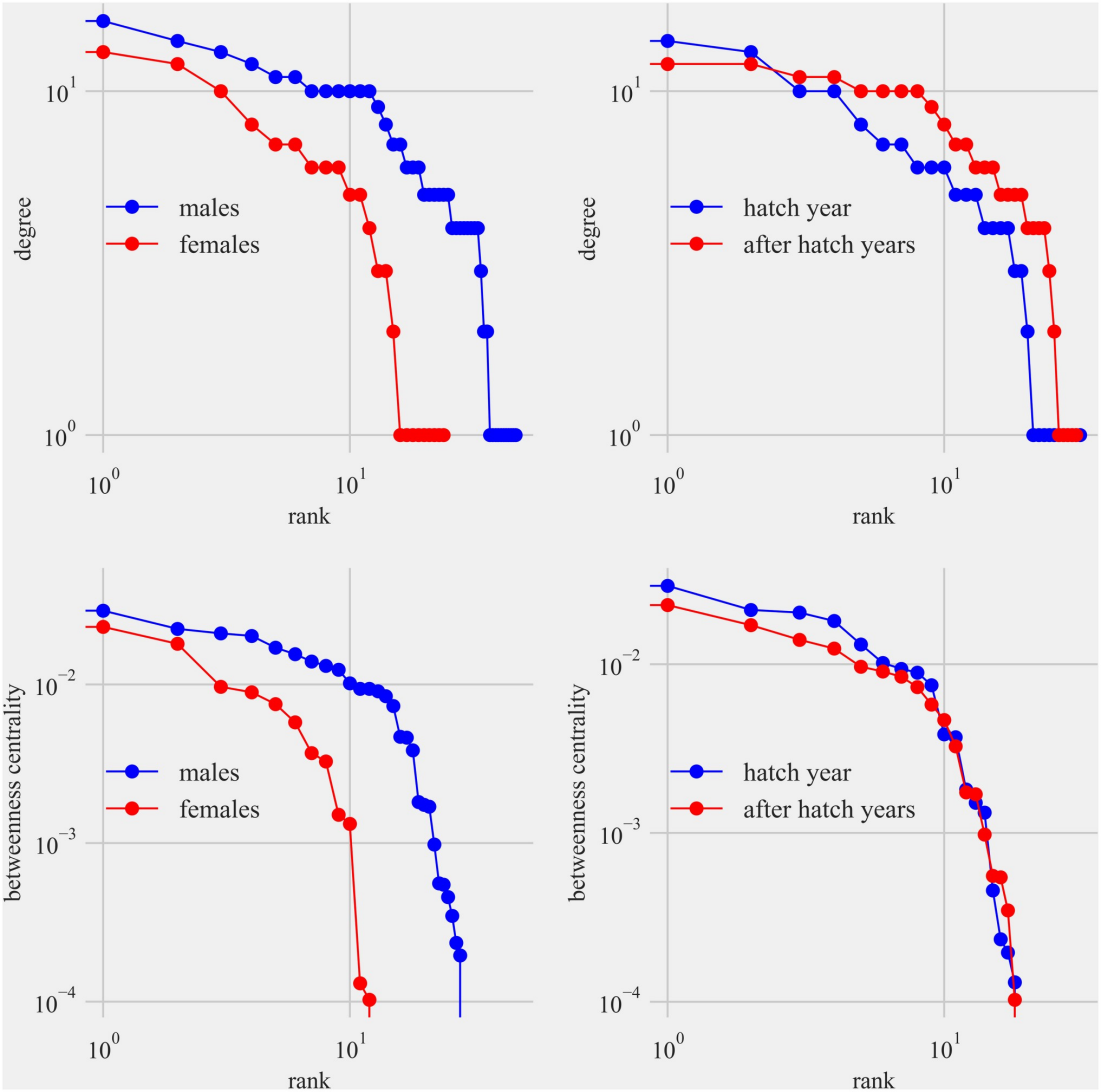
The degree centrality and betweenness centrality of Anna’s hummingbirds shown in two plots at the top and Allen’s hummingbirds at the bottom by sex with the male curve in blue and female curve in red and hatch year in blue and after-hatch year in red. Note that the curves of the betweenness centrality between sexes and two age classes are parallel and not spaced far apart. The distribution of centrality measures of the network (degree and betweenness centrality) showed no significant difference between age and sex of the bird (*p* > 0.05).

Network analysis of the contact network constructed using the interactions detected indicated that there were three separate communities of interacting birds at our study sites despite movement of birds between Sites 1 and 2 (Fig 10). The size of the node is proportional to the degree of the interaction. Edges represent time spent together at the feeding station and the width of the edge width is proportional to the time spent together. Birds on an average interacted with (degree centrality) 7.63 (SD = 5.8, range = 1–16) birds and the birds showed betweenness centrality of 0.004 (SD = 0.007) in the network (Fig 11). After 10,000 permutations the regression analysis showed that the effects of age and sex on the degree and betweenness centrality of the individuals were statistically not significant (*p* > 0.05). Details of the permutations and the regression are presented in S4 and S5 Figs.

## Discussion

The results from this study showed that our RFID equipped feeding station recorded tag data from multiple PIT tagged hummingbirds simultaneously visiting a feeder and allowed us to characterize contact networks in hummingbird populations and quantify feeder visitations. We were able to quantify the patterns in and duration of the feeder visits, along with quantifying the usage of feeders to identify the most accessed feeder at sites with multiple feeders.

Compared to previous studies using RFID technology in hummingbirds, our study was unique in that we described contact networks that exist between hummingbirds visiting feeders. This afforded the ability to study interactions between two hummingbird species that do not normally co-mingle in large numbers in free ranging settings. These birds in our study were assumed to be highly congregated due to resource provisioning in an urbanized setting. Even though our study findings did not highlight any association of sex or age with the centrality of the contact network, we provided an empirical framework for establishing a contact network within hummingbird communities in urbanized habitat [9–11]. Until now, interactions between birds at artificial feeders have been statistically estimated by applying machine learning algorithms to identify clusters of co-feeding events [17]. To the best of our knowledge, this is the first study to quantify actual hummingbird interactions at feeders based on PIT tag detection data. The frequently recorded short interactions detected by our system most likely represented aggressive behavior of individuals defending the feeder [36]. These were considered to be transient interactions. For that reason, the current system would detect both birds only once during such occasions with an interaction time of less than ten seconds. Future studies could confirm this suspicion about the aggressive behavior by incorporating video monitoring. On the other hand, the long interactions were likely from two birds simultaneously visiting the feeding station. However, similarly to the transient interactions, video monitoring could help confirm the birds’ activities.

The RFID equipped feeding station also allowed us to explore the seasonal variation in the daily patterns of feeders visited by Anna’s Hummingbirds. In general, visits to the feeder were most abundant during dawn and dusk hours for the summer season. Our study also provided data that feeder visitation also occurred during night hours by several hummingbirds on sporadic occasions at Study Site 2. Hummingbirds are typically thought to go into a state of torpor at night as a mechanism to conserve energy by lowering their heart and respiration rates [37]. However, torpor in hummingbirds is influenced by the availability of resources [38, 39]. It is also known that nesting females in the advances stages of incubation do not go in the state of torpor [40]. Our findings of night feeder visitations support previous reports [41] that hummingbirds don’t always go into a torpid state at night. Interestingly, the birds feeding at night were two hatch year females and five after-hatch year males at Site 2, mostly during the winter season (January- February) followed by spring (May—June). No activity was seen during summer and fall season between 10 pm to 4 am. Another interesting detail was that Site 2 was the only site in close proximity to street lights. The artificial light source near the feeder could have facilitated night visits, either due to the artificial presence of light [42, 43] or a well-lit feeder station might be perceived by a hummingbird as a “safer” refuge during the night. Video recordings of night activity of hummingbirds were reported in 2012 [44], but to the authors’ knowledge, no PIT tag visitation data for nocturnal feeding activity by hummingbirds exists.

Continuous longitudinal tag detections of marked hummingbirds implied that the RFID equipped feeding station did not deter birds from visiting the instrumented feeders. Based on previous efforts with the RFID equipped feeding stations, we found that hummingbirds would rapidly start using the feeding stations when food resources were limited due to a relatively large carrying capacity of hummingbirds at the site (L. Tell personal observation). The major benefit of using RFID technology, over other methods for marking hummingbirds (such as banding), was the ability to detect birds at feeders without altering hummingbird behavior due to human presence. As shown in earlier studies using RFID tagging in hummingbirds, this technology is advantageous over traditional methods to develop capture-recapture frameworks and estimate demographic parameters [9]. It avoids relying on repeatedly capturing birds for individual identification. Collecting “recapture” information via RFID methods has the significant advantage that birds need not be handled repeatedly, thus reducing risk to the animals, and potentially avoiding “trap shyness”, the tendency of individuals avoiding recapture after being handled. Overall, we found that males returned to stations more often than the females and their number of visits was also higher than females indicating sex variation in the visitation to these artificially provisioned feeders. Our study findings were substantiated by a previous study where males showed more resource guarding behavior and thus were vigilant about keeping other males away from guarded feeders [45]. In contrast, females spent longer periods of time per visit at the feeding stations.

Our estimate of monthly survival probability (0.76) translates to a very low annual survival probability of 0.04, compared to the few available capture-recapture based estimates for other hummingbird species, which are > 0.3 [46–48]. This is likely due to the presence of transient individuals in the hummingbird population, which bias survival estimates [49]. In our study, the simple CJS model used as a proof of concept can easily be modified to account for transiency to yield unbiased survival estimates. The model could further be extended to investigate the effect of diseases on survival probability, on an individual (for example, incorporating signs of diseases at the time of tagging as a model covariate) or a population level (e.g., incorporating disease prevalence in a multi-population analysis of demographic rates). In addition, though not investigated here, an RFID system could conceivably be modified to record visiting frequencies of un-tagged birds, thus facilitating estimation of population size using closed population mark-re-sight models [50]. Linking information on the health status of individual birds, collected during capture and tagging, to these demographic metrics, could allow for the study of the effect of diseases on urban hummingbird populations. Our study highlights that the use of RFID technology to gather data for estimating survival and population parameters offers advantages of collecting large data sets, that require less labor and subject handling compared to banding methods, and this methodology holds promise for future studies.

Another significant outcome of this study was the expansion of the previous description [11] by Brewer et al. for subcutaneous placement of PIT tags in hummingbirds. As a conservative measure, in our study, initial placement of the PIT tags was achieved by using eyelash glue to secure the tag to the skin and feathers of the dorsal aspect of the bird as has been previously described [10]. Disadvantages to this method were the potential for excess glue to come in contact with the primary feathers thus rendering the hummingbird flightless [51] and the suspected short- term nature of tag adherence to the bird’s skin and feathers. Therefore, subsequent to placing the tags externally and minimal tag readings from those birds, small numbers of birds had PITs placed subcutaneously as previously described [11]. These small numbers of birds were monitored for presence at the RFID feeding unit before larger numbers of study birds were tagged. Although the subcutaneous placement of PIT tags has been previously reported, we found that locating the dorsal ridge of the thoracic and lumbar vertebrae is of utmost importance. This anatomical structure can be used to guide tag placement to either side of the bird’s dorsal region. Care was also taken to ensure that the injection needle was inserted in the caudal half of the body so that neither the injection needle nor the tag disrupted the cervicocephalic air sac membranes which would result in subcutaneous emphysema. In our study, we did not see any cases in which the tags disrupted the cervicocephalic air sac membranes at the time of tag placement or when the PIT tagged birds were recaptured during subsequent trapping sessions. Another modification that we made when placing the tags subcutaneously was the use of a single interrupted suture versus surgical glue. The advantage to the suture method was that the feathers at the injection site could be returned to their normal position thus reducing the amount of exposed skin on the bird and minimizing excessive dermal sun exposure. In addition, skin closure at injection site was substantially improved with the use of a single interrupted suture versus surgical glue.

A technological discovery from this study was that equipping the feeding station with two RFID antennas and transceivers did not offer a substantial advantage in data collection over one RFID antenna and transceiver, especially considering the cost difference for two RFID antennas and receivers compared to one per feeding station. Comparison of the data from the two RFID transceivers from the double antenna feeding station showed that the additional top antenna did not offer any overt advantages. For the most part, as long as the tagged hummingbird was inside the feeding station, both antennas of the double antenna feeding station detected the same tag information. Comparing the tag records between the side and top antennas, we observed a few occasions when the side antenna recorded a visit that the top antenna failed to record. This might be due to tagged hummingbirds hovering outside the feeding station near the side antenna and not actually flying into the feeding station. Therefore, we eventually exclusively used single antenna feeding stations at all sites for our study analysis. One thing to note is that an additional antenna could be advantageous in the event of equipment malfunction. If one RFID transceiver or antenna fails, visits by PIT tagged hummingbirds could be recorded by the secondary top RFID transceiver at the feeding station based on our analysis that shows the overall data from the antennas are comparable.

The vast amount of tag data collected by this RFID feeder system was also a factor that was taken into consideration when designing this study. The RFID transceivers have a capacity to (a) continuously record tag reads from an individual bird up to 10 times a second or (b) intermittently record the same tag every 10 seconds for a total of 30 seconds followed one-minute interval readings for the same tag. The RFID transceiver has a maximum capacity of storing only 20,000 detections. Therefore, we set the duty cycle to record the same tag every 10 seconds due to the limited data storage capacity and the excess detections recorded on continuous mode. This enabled us to gather reasonable amounts of data for evaluating the (a) frequency of visitations, (b) amount of time spent at the feeder, and (c) amount of time that birds were simultaneously present at the feeders in multiples of 11 seconds, while avoiding procurement of excessive data. Ideally, it would be advantageous if the RFID transceiver had a capacity to record detections more often than the current duty cycle but less frequently than multiple times a second. This would permit investigators to calculate the exact amount of time spent at the feeder to the second as well as more accurately quantify interactions between birds at the feeders. Previous studies have employed this technique with greater temporal resolution as their transceiver had multiple options of setting the record time for the same tag from 1 second to several hours [11]. With our current system, the time spent at the feeder is being underestimated as we were unable to document the time spent at the feeder after the last tag detection for the visit if it was less than ten seconds due to the reader settings. Hence, we think that the calculated duration of a long visit will always be less than the actual time spent at the feeder. We assume that these long visits, most likely represent hummingbird feeding activity, and are likely to be under estimated in terms of time given the limitations of the current system. Likewise, transient interactions are probably being overestimated. When there are single tag reads from two birds less than 10 seconds apart it is assumed that these tagged birds interacted during that time frame and the hummingbird with the later tag read drove the individual with the earlier tag read away from the feeder. However, in reality they could have missed interacting with each other by a few seconds. Also, we could not statistically evaluate if the data collected from first nine months at Site 1 with a detection interval of 2 minutes differed significantly from the data collected later in the study period when the recording setting was changed to recording the same tag every 10 seconds. This recording setting was changed to collect more accurate measures of tag events. To address this issue, validation would be needed by comparing data from two antennas at the same feeder with different settings.

As pointed out by previous studies, the cost of RFID equipment [11] versus the cost of banding equipment makes data acquisition using RFID technology a continued challenge. However, the cost of an automated system could potentially be justified given the large volume of data acquired using RFID technology, the reduction in human labor, and the advantage of minimal bird handling. Expanding the use of RFID technology will not only allow investigators to study hummingbird behavior at feeders, but factors influencing disease transmission could also be elucidated. In addition, further understanding of local hummingbird movement, as already shown by the movement of birds between Sites 1 & 2, could be gained. Contact networks between hummingbirds in geographically distant areas could also be evaluated thus aiding in understanding disease transmission when the data is tied with disease surveillance data.

The RFID equipped feeding station described in this study could have extensive applications in the ecological exploration of hummingbird populations ranging from understanding population dynamics, basic phenology, local and regional migration patterns, habitat and landscape effects on survivability, and elucidating transmission of infections within populations. Additionally, this system could possibly be used to perform studies that would help to establish recommendations for best feeding practices for hummingbirds and help maintain healthy hummingbird populations living in urban habitats.

## Supporting information

S1 Table Bird activity summary for all three study site locations in Northern (n = 2) and Southern (n = 1) California. This table only includes birds that had at least one tag reading after the passive integrated transponder was placed.
(DOCX)

S1 Fig Activity of PIT tagged Anna’s Hummingbirds (*Calypte anna*) detected between 10 PM to 4 AM for the study period (September 2016- March 2018). This nocturnal activity was only seen at study site 2 in northern California. The shaded portion represents night time. Days preceding and succeeding the night activity are included showing overall activity around the nocturnal activity.
(TIF)

S2 Fig Diel plots of mean hourly female Anna’s Hummingbird visits to the feeding stations at three sites at northern and southern California from September 2016 to March 2018. The bar denotes the mean hourly visits by hummingbirds and black lines show the standard error. All distributions were statistically non-uniform (probability values reported by season).
(TIF)

S3 Fig Diel plots of mean hourly male Anna’s Hummingbird visits to the feeding stations at three sites at northern and southern California from September 2016 to March 2018. The bar denotes the mean hourly visits by hummingbirds and black lines show the standard error. All distributions were statistically non-uniform (probability values reported by season).
(TIF)

S4 Fig Results of permutation-based regression analysis to understand the effect of age and sex on the degree of nodes (individual hummingbirds) in the observed network. Blue lines show the distribution of coefficients after 10,000 permutations. Red lines show original coefficients.
(TIF)

S5 Fig Results of permutation-based regression analysis to understand the effect of age and sex on the betweenness centrality of nodes (individual hummingbirds) in the observed network. Blue lines show the distribution of coefficients after 10,000 permutations. Red lines show original coefficients.
(TIF)

## References

[1] Robb GN, McDonald RA, Chamberlain DE, Bearhop S. Food for thought: supplementary feeding as a driver of ecological change in avian populations. Frontiers in Ecology and the Environment. 2008;6(9):476–84.

[2] Galbraith JA, Beggs JR, Jones DN, Stanley MC. Supplementary feeding restructures urban bird communities. Proc Natl Acad Sci U S A. 2015;112(20):E2648–57. Epub 2015/05/06. 10.1073/pnas.1501489112 25941361 10.1073/pnas.1501489112 PMC4443315

[3] Fuller RA, Warren PH, Armsworth PR, Barbosa O, Gaston KJ. Garden bird feeding predicts the structure of urban avian assemblages. Diversity and Distributions. 2008;14(1):131–7. 10.1111/j.1472-4642.2007.00439.x

[4] Smith WL. Experiential tourism around the world and at home: definitions and standards. International Journal of Services and Standards. 2005;2(1):1–14.

[5] Suarez R. Hummingbird flight: sustaining the highest mass-specific metabolic rates among vertebrates. Experientia. 1992;48(6):565–70. 1612136 10.1007/BF01920240

[6] Bradley CA, Altizer S. Urbanization and the ecology of wildlife diseases. Trends in ecology & evolution. 2007;22(2):95–102. 17113678 10.1016/j.tree.2006.11.001 PMC7114918

[7] Mulvihill RS, Leberman RC. Bird banding at Powdermill, 1985: with a summary of Ruby-throated Hummingbird banding data: Carnegie Museum of Natural History; 1987.

[8] Bonter DN, Bridge ES. Applications of radio frequency identification (RFID) in ornithological research: a review. Journal of Field Ornithology. 2011;82(1):1–10.

[9] Hou L, Verdirame M, Welch KC. Automated tracking of wild hummingbird mass and energetics over multiple time scales using radio frequency identification (RFID) technology. J Avian Biol. 2015;46(1):1–8.

[10] Ibarra V, Araya-Salas M, Tang Y-p, Park C, Hyde A, Wright TF, et al An RFID Based Smart Feeder for Hummingbirds. Sensors. 2015;15(12):31751–61. 10.3390/s151229886 26694402 10.3390/s151229886 PMC4721805

[11] Brewer LW, Redmond CA, Stafford JM, Hatch GE. Marking ruby‐throated hummingbirds with radio frequency identification tags. The Journal of Wildlife Management. 2011;75(7):1664–7.

[12] Sorensen A, van Beest FM, Brook RK. Impacts of wildlife baiting and supplemental feeding on infectious disease transmission risk: a synthesis of knowledge. Preventive veterinary medicine. 2014;113(4):356–63. 10.1016/j.prevetmed.2013.11.010 24365654 10.1016/j.prevetmed.2013.11.010

[13] Civitello DJ, Allman BE, Morozumi C, Rohr JR. Assessing the direct and indirect effects of food provisioning and nutrient enrichment on wildlife infectious disease dynamics. Phil Trans R Soc B. 2018;373(1745). 10.1098/rstb.2017.0101 PMC5883004 29531153

[14] Becker DJ, Hall RJ, Forbes KM, Plowright RK, Altizer S. Anthropogenic resource subsidies and host–parasite dynamics in wildlife. Phil Trans R Soc B. 2018. 10.1098/rstb.2017.0086 PMC5882992 29531141

[15] Strandin T, Babayan SA, Forbes KM. Reviewing the effects of food provisioning on wildlife immunity. Phil Trans R Soc B. 2018;373(1745). 10.1098/rstb.2017.0088 PMC5882994 29531143

[16] Cox DT, Gaston KJ. Human–nature interactions and the consequences and drivers of provisioning wildlife. Phil Trans R Soc B. 2018;373(1745). 10.1098/rstb.2017.0092 PMC5882998 29531147

[17] Adelman JS, Moyers SC, Farine DR, Hawley DM, editors. Feeder use predicts both acquisition and transmission of a contagious pathogen in a North American songbird. Proc R Soc B; 2015: The Royal Society. 10.1098/rspb.2015.1429 PMC4614752 26378215

[18] Craft ME. Infectious disease transmission and contact networks in wildlife and livestock. Phil Trans R Soc B. 2015;370(1669). 10.1098/rstb.2014.0107 PMC4410373 25870393

[19] Brittingham MC, Temple SA. Avian disease and winter bird feeding. The Passenger Pigeon. 1988;50(3):195–203.

[20] Hudson C, Tudor D. Salmonella typhimurium infection in feral birds. Cornell Vet. 1957;47:394–5. 13437691

[21] Docherty DE, Long RIR. Isolation of a poxvirus from a house finch, Carpodacus mexicanus (Müller). Journal of wildlife diseases. 1986;22(3):420–2. 3016351 10.7589/0090-3558-22.3.420

[22] Godoy LA, Dalbeck LS, Tell LA, Woods LW, Colwell RR, Robinson B, et al Characterization of avian poxvirus in Anna's Hummingbird (Calypte anna) in California, USA. Journal of wildlife diseases. 2013;49(4):978–85. 10.7589/2012-09-230 24502725 10.7589/2012-09-230

[23] Godoy LA, Tell LA, Ernest HB. Hummingbird health: pathogens and disease conditions in the family Trochilidae. Journal of ornithology. 2014;155(1):1–12.

[24] Russell SM, Russell RO. The North American banders' manual for banding hummingbirds: North American Banding Council; 2001.

[25] Charette Y, Rousseu F, Mazerolle MJ, Bélisle M, Bélisle M. Tracking Hummingbird foraging movements and patch-use in the wild with passive integrated transponders.

[26] Otis DL, Burnham KP, White GC, Anderson DR. Statistical inference from capture data on closed animal populations. Wildlife monographs. 1978;(62):3–135.

[27] Pollock KH, Nichols JD, Brownie C, Hines JE. Statistical inference for capture-recapture experiments. Wildlife monographs. 1990:3–97.

[28] Lebreton J-D, Burnham KP, Clobert J, Anderson DR. Modeling survival and testing biological hypotheses using marked animals: a unified approach with case studies. Ecological monographs. 1992;62(1):67–118.

[29] Cormack R. Estimates of survival from the sighting of marked animals. Biometrika. 1964;51(3/4):429–38.

[30] Burnham KP, Anderson DR. Model selection and multimodel inference: a practical information-theoretic approach: Springer Science & Business Media; 2003.

[31] Team RC. R: a language and environment for statistical computing. Vienna, Austria: R Foundation for Statistical Computing; 2017. 2017.

[32] Laake JL, Johnson DS, Conn PB. marked: an R package for maximum likelihood and Markov Chain Monte Carlo analysis of capture–recapture data. Methods in Ecology and Evolution. 2013;4(9):885–90.

[33] White GC, Burnham KP. Program MARK: survival estimation from populations of marked animals. Bird study. 1999;46(sup1):S120–S39.

[34] Wilkie D, editor Rayleigh test for randomness of circular data. Applied statistics; 1983: Citeseer.

[35] Jacomy M, Venturini T, Heymann S, Bastian M. ForceAtlas2, a continuous graph layout algorithm for handy network visualization designed for the Gephi software. PloS one. 2014;9(6):e98679 10.1371/journal.pone.0098679 24914678 10.1371/journal.pone.0098679 PMC4051631

[36] Camfield AF. Resource value affects territorial defense by Broad-tailed and Rufous hummingbirds. J Field Ornithol. 2006;77(2):120–5.

[37] Schuchmann K, Krüger K, Prinzinger R. Torpor in hummingbirds. Bonner zoologische Beiträge. 1983;34(1):273.

[38] Hiebert SM. Seasonal differences in the response of Rufous Hummingbirds to food restriction—body mass and the use of torpor. Condor. 1991;93(3):526–37.

[39] Bartholomew GA, Howell TR, Cade TJ. Torpidity in the white -throated swift, Anna hummingbird, and poor-will. Condor. 1957;59((3)):145–55.

[40] Lasiewski RC. Oxygen consumption of torpid, resting, active and flying hummingbirds. Physiol Zool. 1963;36((2)):122–40.

[41] Powers DR, Brown AR, Van Hook JA. Influence of normal daytime fat deposition on laboratory measurements of torpor use in territorial versus nonterritorial hummingbirds. Physiological and Biochemical Zoology. 2003;76(3):389–97. 10.1086/374286 12905125 10.1086/374286

[42] Hainsworth FR, Collins BG, Wolf LL. The function of torpor in hummingbirds. Physiological Zoology. 1977;50(3):215–22.

[43] Goertz JW, Morris AS, Morris SM. Ruby-throated Hummingbirds feed at night with the aid of artificial light. The Wilson Bulletin. 1980:398–9.

[44] Dale T. Do Hummingbirds Fly at Night? [Youtube Video]. 2012. https://www.youtube.com/watch?v=nOvBQntDXuo.

[45] Gill FB. Trapline foraging by hermit hummingbirds: competition for an undefended, renewable resource. Ecology. 1988;69(6):1933–42.

[46] Rodrigues LdC, Martins FI, Rodrigues M. Survival of a mountaintop hummingbird, the Hyacinth Visorbearer Augastes scutatus, in southeastern Brazil. Acta ornithologica. 2013;48(2):211–9.

[47] Blake JG, Loiselle BA. Estimates of apparent survival rates for forest birds in eastern Ecuador. Biotropica. 2008;40(4):485–93. 10.1371/journal.pone.0081028 PMC3846669 24312519

[48] Hilton B Jr, Miller MW. Annual survival and recruitment in a ruby-throated hummingbird population, excluding the effect of transient individuals. Condor. 2003:54–62.

[49] Pradel R, Hines JE, Lebreton J-D, Nichols JD. Capture-recapture survival models taking account of transients. Biometrics. 1997:60–72.

[50] McClintock BT, White GC, Antolin MF, Tripp DW. Estimating abundance using mark–resight when sampling is with replacement or the number of marked individuals is unknown. Biometrics. 2009;65(1):237–46. 10.1111/j.1541-0420.2008.01047.x 18479484 10.1111/j.1541-0420.2008.01047.x

[51] Wells DJ. Ecological correlates of hovering flight of hummingbirds. Journal of Experimental Biology. 1993;178(1):59–70.

