# Correction: Use of RFID technology to characterize feeder visitations and contact network of hummingbirds in urban habitats

**DOI:** 10.1371/journal.pone.0211254

**Published:** 2019-01-17

**Authors:** Ruta R. Bandivadekar, Pranav S. Pandit, Rahel Sollmann, Michael J. Thomas, Scott M. Logan, Jennifer C. Brown, A. Peter Klimley, Lisa A. Tell

Fig 5 is incorrect. The authors have provided a corrected version here.

**Fig 5:**
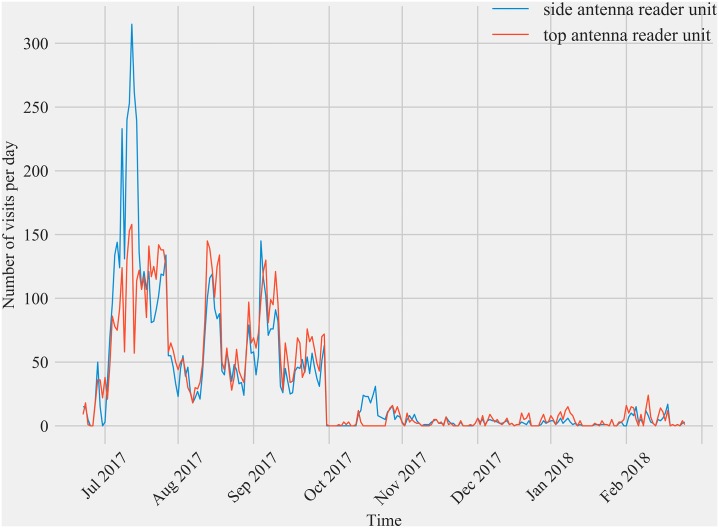
Comparison of the daily detections from the two different antennas at the double antenna feeding station transceiver at Site 2 over time. The top antenna for this feeding station was deployed in May 2017 and discontinued in January 2018 for data collection. Overall, the total number of visits detected by the side antenna exceeded the number of visits for the top antenna but not enough to warrant the addition of a second antenna. Both antennas detected the presence of the same number of individual birds.
