# Supplementary material for: Use of RFID technology to characterize feeder visitations and contact network of hummingbirds in urban habitats
Source: PLoS One. 2018 Dec 12;13(12):e0208057. doi: 10.1371/journal.pone.0208057 (PMC6291107; doi:10.1371/journal.pone.0208057)
Supplement: S1 Table — This table only includes birds that had at least one tag reading after the passive integrated transponder was placed. (DOCX) [file pone.0208057.s001.docx]

**Supporting Information**

**S1 Table. Bird activity summary for all three study site locations in Northern (n=2) and Southern (n=1) California. This table only includes birds that had at least one tag reading after the passive integrated transponder was placed*.***

| Bird number | Location Id | Species | Age | Sex | Hex Tag Number | Date Tagged | Date first Detected | Date last Detected | First detected after tagged (days) | Total Observation Period = Last Detected - tagged Date | Number of days the bird was detected at the feeder | Total duration of time spent at the feeder (seconds) | Total duration of time spent at the feeder: Mean (seconds) | Total duration of time spent at the feeder: Median (seconds) | Shortest Visit (seconds) | Longest Visit (Seconds) |
| --- | --- | --- | --- | --- | --- | --- | --- | --- | --- | --- | --- | --- | --- | --- | --- | --- |
| Bird 1 | Site 1 | ANHU | HY | F | 3D6.00184967A1 | 9/23/2016 | 9/25/2016 | 12/11/2017 | 2 | 444 | 90 | 4077 | 6.5 | 0 | 0 | 143 |
| Bird 2 | Site 2 | ANHU | AHY | F | 3D6.00184967A2 | 5/19/2017 | 5/20/2017 | 3/29/2018 | 1 | 314 | 95 | 16473 | 19.3 | 0 | 0 | 142 |
| Bird 3 | Site 1 | ANHU | AHY | F | 3D6.00184967A3 | 9/23/2016 | 11/10/2016 | 11/28/2016 | 48 | 66 | 2 | 0 | 0.0 | 0 | 0 | 0 |
| Bird 4 | Site 2 | ANHU | HY | M | 3D6.00184967A4 | 7/11/2017 | 7/11/2017 | 3/31/2018 | 0 | 263 | 219 | 44335 | 15.0 | 10 | 0 | 153 |
| Bird 5 | Site 2 | ANHU | HY | M | 3D6.00184967A6 | 6/5/2017 | 6/8/2017 | 6/24/2017 | 3 | 19 | 12 | 1776 | 6.3 | 0 | 0 | 110 |
| Bird 6 | Site 2 | ANHU | HY | F | 3D6.00184967A7 | 6/1/2017 | 6/1/2017 | 6/28/2017 | 0 | 27 | 28 | 16148 | 7.6 | 0 | 0 | 187 |
| Bird 7 | Site 2 | ANHU | HY | M | 3D6.00184967A8 | 5/26/2017 | 5/26/2017 | 6/11/2017 | 0 | 16 | 15 | 1517 | 5.2 | 0 | 0 | 44 |
| Bird 8 | Site 1 | ANHU | HY | M | 3D6.00184967A9 | 9/9/2016 | 9/30/2016 | 10/23/2016 | 21 | 44 | 24 | 0 | 0.0 | 0 | 0 | 0 |
| Bird 9 | Site 1 | ANHU | HY | F | 3D6.00184967AB | 9/9/2016 | 10/4/2016 | 10/21/2016 | 25 | 42 | 17 | 0 | 0.0 | 0 | 0 | 0 |
| Bird 10 | Site 2 | ANHU | AHY | M | 3D6.00184967AC | 5/26/2017 | 5/26/2017 | 5/29/2017 | 0 | 3 | 2 | 0 | 0.0 | 0 | 0 | 0 |
| Bird 11 | Site 2 | ANHU | AHY | M | 3D6.00184967AD | 5/19/2017 | 5/19/2017 | 3/31/2018 | 0 | 316 | 63 | 13159 | 10.3 | 0 | 0 | 219 |
| Bird 12 | Site 1 | ANHU | AHY | F | 3D6.00184967AE | 9/9/2016 | 9/14/2016 | 9/14/2016 | 5 | 5 | 1 | 0 | 0.0 | 0 | 0 | 0 |
| Bird 13 | Site 2 | ANHU | AHY | M | 3D6.00184967AF | 5/19/2017 | 5/19/2017 | 3/31/2018 | 0 | 316 | 139 | 27212 | 16.1 | 11 | 0 | 252 |
| Bird 14 | Site 1 | ANHU | AHY | M | 3D6.00184967B0 | 9/2/2016 | 10/1/2016 | 1/14/2017 | 29 | 134 | 64 | 0 | 0.0 | 0 | 0 | 0 |
| Bird 15 | Site 1 | ANHU | AHY | F | 3D6.00184967B5 | 9/23/2016 | 10/11/2016 | 10/21/2016 | 18 | 28 | 5 | 0 | 0.0 | 0 | 0 | 0 |
| Bird 16 | Site 1 | ANHU | HY | F | 3D6.00184967B6 | 7/14/2017 | 7/14/2017 | 7/16/2017 | 0 | 2 | 2 | 393 | 7.9 | 0 | 0 | 66 |
| Bird 17 | Site 2 | ANHU | AHY | M | 3D6.00184967B7 | 5/19/2017 | 5/19/2017 | 5/20/2017 | 0 | 1 | 2 | 151 | 25.2 | 15 | 3 | 74 |
| Bird 18 | Site 2 | ANHU | AHY | F | 3D6.00184967B8 | 7/11/2017 | 7/11/2017 | 3/31/2018 | 0 | 263 | 111 | 24485 | 19.2 | 10 | 0 | 143 |
| Bird 19 | Site 1 | ANHU | HY | F | 3D6.00184967B9 | 9/9/2016 | 9/10/2016 | 10/25/2017 | 1 | 411 | 123 | 16188 | 10.3 | 0 | 0 | 143 |
| Bird 20 | Site 2 | ANHU | HY | M | 3D6.00184967BA | 5/30/2017 | 6/4/2017 | 6/8/2017 | 5 | 9 | 3 | 110 | 7.9 | 0 | 0 | 33 |
| Bird 21 | Site 2 | ANHU | HY | F | 3D6.00184967BC | 5/30/2017 | 5/30/2017 | 8/31/2017 | 0 | 93 | 94 | 59922 | 24.2 | 0 | 0 | 286 |
| Bird 22 | Site 1 | ANHU | AHY | M | 3D6.00184967BE | 7/14/2017 | 7/14/2017 | 8/6/2017 | 0 | 23 | 5 | 97 | 2.4 | 0 | 0 | 44 |
| Bird 23 | Site 2 | ANHU | AHY | M | 3D6.00184967BF | 7/11/2017 | 7/11/2017 | 3/31/2018 | 0 | 263 | 202 | 36264 | 11.7 | 10 | 0 | 109 |
| Bird 24 | Site 2 | ANHU | AHY | M | 3D6.00184967C1 | 8/14/2017 | 2/13/2018 | 2/13/2018 | 183 | 183 | 1 | 108 | 27.0 | 26.5 | 0 | 55 |
| Bird 25 | Site 1 | ANHU | HY | M | 3D6.00184967C3 | 9/9/2016 | 9/26/2016 | 12/7/2017 | 17 | 454 | 134 | 6880 | 3.9 | 0 | 0 | 153 |
| Bird 26 | Site 1 | ANHU | HY | F | 3D6.00184967C4 | 9/9/2016 | 2/25/2017 | 5/23/2017 | 169 | 256 | 46 | 0 | 0.0 | 0 | 0 | 0 |
| Bird 27 | Site 1 | ANHU | AHY | M | 3D6.00184967C5 | 9/27/2017 | 10/10/2017 | 10/10/2017 | 13 | 13 | 1 | 33 | 33.0 | 33 | 33 | 33 |
| Bird 28 | Site 1 | ANHU | HY | M | 3D6.00184967C6 | 9/9/2016 | 9/9/2016 | 9/13/2016 | 0 | 4 | 4 | 0 | 0.0 | 0 | 0 | 0 |
| Bird 29 | Site 2 | ANHU | HY | F | 3D6.00184967C8 | 5/26/2017 | 5/28/2017 | 5/31/2017 | 2 | 5 | 4 | 225 | 2.3 | 0 | 0 | 55 |
| Bird 30 | Site 1 | ANHU | HY | F | 3D6.00184967CB | 7/17/2017 | 7/17/2017 | 3/15/2018 | 0 | 241 | 81 | 6968 | 10.2 | 0 | 0 | 175 |
| Bird 31 | Site 2 | ANHU | HY | M | 3D6.00184967CC | 8/7/2017 | 8/7/2017 | 9/8/2017 | 0 | 32 | 28 | 4719 | 13.3 | 10 | 0 | 121 |
| Bird 32 | Site 2 | ANHU | HY | M | 3D6.00184967CE | 8/7/2017 | 8/8/2017 | 8/19/2017 | 1 | 12 | 3 | 0 | 0.0 | 0 | 0 | 0 |
| Bird 33 | Site 1 | ANHU | AHY | M | 3D6.00184967D0 | 6/21/2017 | 6/23/2017 | 6/28/2017 | 2 | 7 | 2 | 0 | 0.0 | 0 | 0 | 0 |
| Bird 34 | Site 2 | ANHU | HY | M | 3D6.00184967D1 | 7/11/2017 | 7/18/2017 | 8/9/2017 | 7 | 29 | 21 | 4814 | 9.8 | 10 | 0 | 54 |
| Bird 35 | Site 1 | ANHU | HY | F | 3D6.00184967D2 | 7/14/2017 | 7/14/2017 | 7/24/2017 | 0 | 10 | 4 | 10 | 1.7 | 0 | 0 | 10 |
| Bird 36 | Site 1 | ANHU | AHY | F | 3D6.00184967D3 | 9/23/2016 | 9/23/2016 | 9/23/2016 | 0 | 0 | 1 | 0 | 0.0 | 0 | 0 | 0 |
| Bird 37 | Site 2 | ANHU | HY | M | 3D6.00184967D4 | 7/11/2017 | 7/11/2017 | 7/11/2017 | 0 | 0 | 1 | 0 | 0.0 | 0 | 0 | 0 |
| Bird 38 | Site 2 | ANHU | HY | M | 3D6.00184967D5 | 5/23/2017 | 5/23/2017 | 5/26/2017 | 0 | 3 | 4 | 467 | 8.8 | 0 | 0 | 77 |
| Bird 39 | Site 1 | ANHU | HY | F | 3D6.00184967D6 | 9/23/2016 | 12/11/2017 | 2/2/2018 | 444 | 497 | 3 | 21 | 3.5 | 0 | 0 | 21 |
| Bird 40 | Site 1 | ANHU | AHY | M | 3D6.00184967D7 | 9/23/2016 | 9/23/2016 | 3/31/2018 | 0 | 554 | 289 | 1331 | 0.3 | 0 | 0 | 22 |
| Bird 41 | Site 2 | ANHU | HY | F | 3D6.00184967D9 | 5/30/2017 | 8/18/2017 | 11/28/2017 | 80 | 182 | 41 | 4171 | 21.5 | 11 | 0 | 143 |
| Bird 42 | Site 1 | ANHU | HY | F | 3D6.00184967DA | 8/28/2017 | 8/30/2017 | 8/30/2017 | 2 | 2 | 1 | 10 | 1.7 | 0 | 0 | 10 |
| Bird 43 | Site 1 | ANHU | AHY | M | 3D6.00184967DB | 10/21/2016 | 10/21/2016 | 10/27/2016 | 0 | 6 | 5 | 0 | 0.0 | 0 | 0 | 0 |
| Bird 44 | Site 1 | ANHU | HY | M | 3D6.00184967DD | 6/21/2017 | 11/10/2017 | 1/20/2018 | 142 | 213 | 14 | 1602 | 22.6 | 0 | 0 | 186 |
| Bird 45 | Site 2 | ANHU | AHY | F | 3D6.00184967E0 | 7/11/2017 | 7/11/2017 | 7/11/2017 | 0 | 0 | 1 | 0 | 0.0 | 0 | 0 | 0 |
| Bird 46 | Site 1 | ANHU | HY | M | 3D6.00184967E1 | 10/10/2017 | 10/13/2017 | 10/13/2017 | 3 | 3 | 1 | 0 | 0.0 | 0 | 0 | 0 |
| Bird 47 | Site 1 | ANHU | AHY | M | 3D6.00184967E2 | 10/10/2017 | 2/3/2018 | 3/31/2018 | 116 | 172 | 39 | 2982 | 7.9 | 0 | 0 | 110 |
| Bird 48 | Site 2 | ANHU | AHY | M | 3D6.00184967E3 | 5/26/2017 | 5/28/2017 | 6/12/2017 | 2 | 17 | 2 | 0 | 0.0 | 0 | 0 | 0 |
| Bird 49 | Site 1 | ANHU | AHY | F | 3D6.00184967E4 | 10/10/2017 | 10/11/2017 | 10/26/2017 | 1 | 16 | 5 | 0 | 0.0 | 0 | 0 | 0 |
| Bird 50 | Site 2 | ANHU | AHY | F | 3D6.00184967E6 | 6/2/2017 | 6/3/2017 | 6/15/2017 | 1 | 13 | 13 | 4635 | 10.5 | 0 | 0 | 66 |
| Bird 51 | Site 2 | ANHU | AHY | F | 3D6.00184967E7 | 6/1/2017 | 6/1/2017 | 3/31/2018 | 0 | 303 | 115 | 30235 | 19.3 | 11 | 0 | 132 |
| Bird 52 | Site 2 | ANHU | AHY | F | 3D6.00184967E9 | 7/11/2017 | 3/23/2018 | 3/26/2018 | 255 | 258 | 2 | 0 | 0.0 | 0 | 0 | 0 |
| Bird 53 | Site 2 | ANHU | HY | M | 3D6.00184967ED | 7/11/2017 | 7/11/2017 | 8/16/2017 | 0 | 36 | 37 | 16455 | 12.4 | 10 | 0 | 132 |
| Bird 54 | Site 1 | ANHU | HY | F | 3D6.00184967EF | 7/14/2017 | 7/16/2017 | 3/31/2018 | 2 | 260 | 52 | 1176 | 0.9 | 0 | 0 | 32 |
| Bird 55 | Site 2 | ANHU | HY | M | 3D6.00184967F1 | 5/30/2017 | 5/30/2017 | 8/9/2017 | 0 | 71 | 48 | 6465 | 6.7 | 0 | 0 | 77 |
| Bird 56 | Site 1 | ANHU | HY | M | 3D6.00184967F4 | 7/14/2017 | 7/14/2017 | 7/27/2017 | 0 | 13 | 14 | 3262 | 7.4 | 0 | 0 | 99 |
| Bird 57 | Site 1 | ANHU | HY | F | 3D6.00184967F5 | 7/14/2017 | 7/31/2017 | 8/3/2017 | 17 | 20 | 2 | 0 | 0.0 | 0 | 0 | 0 |
| Bird 58 | Site 2 | ANHU | HY | M | 3D6.00184967F7 | 7/11/2017 | 7/12/2017 | 8/14/2017 | 1 | 34 | 12 | 1484 | 31.6 | 22 | 0 | 121 |
| Bird 59 | Site 2 | ANHU | AHY | M | 3D6.00184967F8 | 6/1/2017 | 3/7/2018 | 3/31/2018 | 279 | 303 | 25 | 9269 | 15.0 | 11 | 0 | 132 |
| Bird 60 | Site 1 | ANHU | AHY | M | 3D6.00184967FE | 10/10/2017 | 10/11/2017 | 3/28/2018 | 1 | 169 | 78 | 3709 | 5.5 | 0 | 0 | 198 |
| Bird 61 | Site 1 | ANHU | AHY | F | 3D6.0018496801 | 7/14/2017 | 8/24/2017 | 9/3/2017 | 41 | 51 | 5 | 74 | 5.7 | 0 | 0 | 32 |
| Bird 62 | Site 1 | ANHU | HY | F | 3D6.0018496802 | 10/10/2017 | 2/1/2018 | 2/5/2018 | 114 | 118 | 3 | 54 | 7.7 | 0 | 0 | 33 |
| Bird 63 | Site 3 | ANHU | UNK | M | 3D6.001881F730 | 11/29/2017 | 12/24/2017 | 1/31/2018 | 25 | 63 | 2 | 0 | 0.0 | 0 | 0 | 0 |
| Bird 64 | Site 3 | ANHU | UNK | M | 3D6.001881F733 | 10/15/2017 | 1/14/2018 | 2/19/2018 | 91 | 127 | 5 | 0 | 0.0 | 0 | 0 | 0 |
| Bird 65 | Site 3 | ANHU | UNK | F | 3D6.001881F736 | 11/29/2017 | 12/8/2017 | 1/28/2018 | 9 | 60 | 7 | 0 | 0.0 | 0 | 0 | 0 |
| Bird 66 | Site 3 | ANHU | UNK | M | 3D6.001881F73A | 11/30/2017 | 1/1/2018 | 1/8/2018 | 32 | 39 | 2 | 228 | 16.3 | 0 | 0 | 154 |
| Bird 67 | Site 3 | ALHU | UNK | M | 3D6.001881F743 | 10/16/2017 | 11/5/2017 | 2/9/2018 | 20 | 116 | 4 | 10 | 1.0 | 0 | 0 | 10 |
| Bird 68 | Site 3 | ANHU | UNK | M | 3D6.001881F758 | 11/30/2017 | 12/24/2017 | 3/26/2018 | 24 | 116 | 14 | 0 | 0.0 | 0 | 0 | 0 |
| Bird 69 | Site 3 | ANHU | UNK | M | 3D6.001881F761 | 10/16/2017 | 10/20/2017 | 11/15/2017 | 4 | 30 | 6 | 0 | 0.0 | 0 | 0 | 0 |
| Bird 70 | Site 3 | ANHU | AHY | M | 3D6.001881F76C | 10/16/2017 | 12/30/2017 | 3/31/2018 | 75 | 166 | 24 | 42 | 0.5 | 0 | 0 | 11 |
| Bird 71 | Site 3 | ANHU | UNK | M | 3D6.001881F775 | 10/15/2017 | 2/16/2018 | 3/15/2018 | 124 | 151 | 4 | 0 | 0.0 | 0 | 0 | 0 |
| Bird 72 | Site 3 | ALHU | UNK | F | 3D6.001881F776 | 11/30/2017 | 12/2/2017 | 12/2/2017 | 2 | 2 | 1 | 109 | 12.1 | 0 | 0 | 76 |
| Bird 73 | Site 3 | ANHU | UNK | M | 3D6.001881F777 | 10/15/2017 | 11/13/2017 | 12/23/2017 | 29 | 69 | 2 | 0 | 0.0 | 0 | 0 | 0 |
| Bird 74 | Site 3 | ANHU | UNK | F | 3D6.001881F780 | 10/15/2017 | 11/10/2017 | 11/10/2017 | 26 | 26 | 1 | 0 | 0.0 | 0 | 0 | 0 |
| Bird 75 | Site 3 | ANHU | UNK | F | 3D6.001881F782 | 11/30/2017 | 2/21/2018 | 2/23/2018 | 83 | 85 | 3 | 10 | 1.4 | 0 | 0 | 10 |
| Bird 76 | Site 3 | ANHU | UNK | M | 3D6.001881F783 | 11/30/2017 | 12/22/2017 | 3/25/2018 | 22 | 115 | 11 | 0 | 0.0 | 0 | 0 | 0 |
| Bird 77 | Site 3 | ALHU | HY | M | 3D6.001881F784 | 11/29/2017 | 2/12/2018 | 3/31/2018 | 75 | 122 | 23 | 44 | 0.2 | 0 | 0 | 11 |
| Bird 78 | Site 3 | ANHU | HY | M | 3D6.001881F788 | 10/15/2017 | 1/13/2018 | 3/11/2018 | 90 | 147 | 10 | 0 | 0.0 | 0 | 0 | 0 |
| Bird 79 | Site 3 | ANHU | HY | M | 3D6.001881F78A | 10/15/2017 | 12/5/2017 | 12/9/2017 | 51 | 55 | 2 | 0 | 0.0 | 0 | 0 | 0 |
| Bird 80 | Site 3 | ANHU | UNK | F | 3D6.1D593D45B3 | 11/29/2017 | 2/6/2018 | 2/6/2018 | 69 | 69 | 1 | 0 | 0.0 | 0 | 0 | 0 |
| Bird 81 | Site 3 | ALHU | UNK | M | 3D6.1D593D45B6 | 11/30/2017 | 1/14/2018 | 1/26/2018 | 45 | 57 | 4 | 0 | 0.0 | 0 | 0 | 0 |
| Bird 82 | Site 3 | ALHU | UNK | M | 3D6.1D593D45B9 | 11/29/2017 | 3/31/2018 | 3/31/2018 | 122 | 122 | 1 | 0 | 0.0 | 0 | 0 | 0 |
| Bird 83 | Site 3 | ALHU | AHY | M | 3D6.1D593D45C7 | 2/25/2018 | 2/26/2018 | 2/26/2018 | 1 | 1 | 1 | 0 | 0.0 | 0 | 0 | 0 |
| Bird 84 | Site 3 | ALHU | AHY | M | 3D6.1D593D45C9 | 2/25/2018 | 2/25/2018 | 3/11/2018 | 0 | 14 | 15 | 2165 | 2.5 | 0 | 0 | 87 |
| Bird 85 | Site 3 | ANHU | UNK | F | 3D6.1D593D45CB | 10/16/2017 | 11/1/2017 | 1/25/2018 | 16 | 101 | 4 | 0 | 0.0 | 0 | 0 | 0 |
| Bird 86 | Site 3 | ALHU | AHY | M | 3D6.1D593D45CE | 2/25/2018 | 2/26/2018 | 2/26/2018 | 1 | 1 | 1 | 0 | 0.0 | 0 | 0 | 0 |
| Bird 87 | Site 3 | ANHU | AHY | F | 3D6.1D593D45CF | 2/25/2018 | 2/28/2018 | 3/31/2018 | 3 | 34 | 32 | 33 | 0.2 | 0 | 0 | 11 |
| Bird 88 | Site 3 | ALHU | UNK | M | 3D6.1D593D45D0 | 11/29/2017 | 11/29/2017 | 3/9/2018 | 0 | 100 | 97 | 3332 | 1.5 | 0 | 0 | 77 |
| Bird 89 | Site 3 | ANHU | UNK | M | 3D6.1D593D45D1 | 11/30/2017 | 12/22/2017 | 12/27/2017 | 22 | 27 | 3 | 0 | 0.0 | 0 | 0 | 0 |
| Bird 90 | Site 3 | ALHU | AHY | M | 3D6.1D593D45D2 | 2/26/2018 | 3/12/2018 | 3/12/2018 | 14 | 14 | 1 | 0 | 0.0 | 0 | 0 | 0 |
| Bird 91 | Site 3 | ALHU | UNK | M | 3D6.1D593D45D3 | 11/29/2017 | 12/22/2017 | 12/22/2017 | 23 | 23 | 1 | 130 | 26.0 | 10 | 0 | 109 |
| Bird 92 | Site 3 | ALHU | AHY | M | 3D6.1D593D45D4 | 2/25/2018 | 3/7/2018 | 3/8/2018 | 10 | 11 | 2 | 0 | 0.0 | 0 | 0 | 0 |
| Bird 93 | Site 3 | ANHU | UNK | F | 3D6.1D593D45D7 | 10/15/2017 | 10/20/2017 | 11/3/2017 | 5 | 19 | 12 | 848 | 3.8 | 0 | 0 | 99 |
| Bird 94 | Site 3 | ALHU | UNK | F | 3D6.1D593D45DA | 11/30/2017 | 12/1/2017 | 1/30/2018 | 1 | 61 | 2 | 0 | 0.0 | 0 | 0 | 0 |
| Bird 95 | Site 3 | ALHU | UNK | M | 3D6.1D593D45DB | 11/30/2017 | 3/14/2018 | 3/16/2018 | 104 | 106 | 2 | 0 | 0.0 | 0 | 0 | 0 |
| Bird 96 | Site 3 | ALHU | UNK | M | 3D6.1D593D45DF | 10/16/2017 | 12/6/2017 | 3/30/2018 | 51 | 165 | 46 | 0 | 0.0 | 0 | 0 | 0 |
| Bird 97 | Site 3 | ANHU | HY | M | 3D6.1D593D45E1 | 11/29/2017 | 2/21/2018 | 2/21/2018 | 84 | 84 | 1 | 0 | 0.0 | 0 | 0 | 0 |
| Bird 98 | Site 3 | ALHU | AHY | M | 3D6.1D593D45E2 | 2/26/2018 | 3/8/2018 | 3/8/2018 | 10 | 10 | 1 | 0 | 0.0 | 0 | 0 | 0 |
| Bird 99 | Site 3 | ALHU | AHY | F | 3D6.1D593D45E4 | 2/26/2018 | 3/11/2018 | 3/25/2018 | 13 | 27 | 2 | 0 | 0.0 | 0 | 0 | 0 |
| Bird 100 | Site 3 | ALHU | UNK | M | 3D6.1D593D45E5 | 11/30/2017 | 12/5/2017 | 12/25/2017 | 5 | 25 | 3 | 0 | 0.0 | 0 | 0 | 0 |
| Bird 101 | Site 3 | ANHU | AHY | M | 3D6.1D593D45E6 | 2/26/2018 | 2/26/2018 | 3/31/2018 | 0 | 33 | 34 | 1221 | 1.0 | 0 | 0 | 33 |
| Bird 102 | Site 3 | ALHU | AHY | F | 3D6.1D593D45E7 | 2/25/2018 | 3/2/2018 | 3/12/2018 | 5 | 15 | 2 | 0 | 0.0 | 0 | 0 | 0 |
| Bird 103 | Site 3 | ANHU | HY | M | 3D6.1D593D45E9 | 10/15/2017 | 10/16/2017 | 11/12/2017 | 1 | 28 | 2 | 66 | 33.0 | 33 | 0 | 66 |
| Bird 104 | Site 3 | ANHU | UNK | M | 3D6.1D593D45EB | 10/15/2017 | 12/23/2017 | 3/28/2018 | 69 | 164 | 61 | 462 | 0.9 | 0 | 0 | 65 |
| Bird 105 | Site 3 | ANHU | HY | M | 3D6.1D593D45EF | 10/15/2017 | 12/1/2017 | 12/1/2017 | 47 | 47 | 1 | 0 | 0.0 | 0 | 0 | 0 |
| Bird 106 | Site 3 | ANHU | HY | F | 3D6.1D593D45F1 | 10/15/2017 | 12/25/2017 | 3/31/2018 | 71 | 167 | 79 | 3806 | 2.0 | 0 | 0 | 197 |
| Bird 107 | Site 3 | ALHU | AHY | M | 3D6.1D593D45F2 | 2/26/2018 | 2/26/2018 | 3/24/2018 | 0 | 26 | 27 | 1517 | 1.2 | 0 | 0 | 66 |
| Bird 108 | Site 3 | ANHU | AHY | M | 3D6.1D593D45F3 | 2/25/2018 | 2/27/2018 | 2/27/2018 | 2 | 2 | 1 | 0 | 0.0 | 0 | 0 | 0 |
| Bird 109 | Site 3 | ALHU | UNK | M | 3D6.1D593D45F7 | 11/30/2017 | 12/2/2017 | 12/2/2017 | 2 | 2 | 1 | 0 | 0.0 | 0 | 0 | 0 |
| Bird 110 | Site 3 | ANHU | AHY | F | 3D6.1D593D45F9 | 2/25/2018 | 3/25/2018 | 3/26/2018 | 28 | 29 | 2 | 0 | 0.0 | 0 | 0 | 0 |
| Bird 111 | Site 3 | ANHU | HY | M | 3D6.1D593D45FA | 10/15/2017 | 10/26/2017 | 2/28/2018 | 11 | 136 | 63 | 845 | 2.0 | 0 | 0 | 99 |
| Bird 112 | Site 3 | ALHU | UNK | M | 3D6.1D593D45FB | 11/29/2017 | 1/20/2018 | 3/29/2018 | 52 | 120 | 2 | 0 | 0.0 | 0 | 0 | 0 |
| Bird 113 | Site 3 | ANHU | UNK | M | 3D6.1D593D45FF | 10/15/2017 | 11/10/2017 | 3/31/2018 | 26 | 167 | 96 | 13373 | 2.2 | 0 | 0 | 143 |
| Bird 114 | Site 3 | ANHU | HY | M | 3D6.1D593D4601 | 10/15/2017 | 12/23/2017 | 2/23/2018 | 69 | 131 | 3 | 0 | 0.0 | 0 | 0 | 0 |
| Bird 115 | Site 3 | ALHU | UNK | F | 3D6.1D593D4603 | 11/29/2017 | 2/23/2018 | 2/23/2018 | 86 | 86 | 1 | 0 | 0.0 | 0 | 0 | 0 |
| Bird 116 | Site 3 | ANHU | UNK | M | 3D6.1D593D4604 | 10/15/2017 | 12/9/2017 | 1/17/2018 | 55 | 94 | 3 | 0 | 0.0 | 0 | 0 | 0 |
| Bird 117 | Site 3 | ANHU | AHY | F | 3D6.1D593D4606 | 2/26/2018 | 2/26/2018 | 3/17/2018 | 0 | 19 | 6 | 0 | 0.0 | 0 | 0 | 0 |
| Bird 118 | Site 3 | ALHU | UNK | F | 3D6.1D593D4607 | 11/29/2017 | 1/16/2018 | 3/25/2018 | 48 | 116 | 2 | 0 | 0.0 | 0 | 0 | 0 |
| Bird 119 | Site 3 | ALHU | UNK | F | 3D6.1D593D4609 | 11/29/2017 | 3/18/2018 | 3/26/2018 | 109 | 117 | 2 | 11 | 2.8 | 0 | 0 | 11 |
| Bird 120 | Site 3 | ANHU | UNK | M | 3D6.1D593D460A | 11/29/2017 | 2/4/2018 | 2/4/2018 | 67 | 67 | 1 | 0 | 0.0 | 0 | 0 | 0 |
| Bird 121 | Site 3 | ALHU | UNK | M | 3D6.1D593D460B | 11/29/2017 | 11/29/2017 | 3/29/2018 | 0 | 120 | 9 | 21 | 1.9 | 0 | 0 | 21 |
| Bird 122 | Site 3 | ALHU | AHY | M | 3D6.1D593D460D | 2/26/2018 | 2/26/2018 | 2/26/2018 | 0 | 0 | 1 | 0 | 0.0 | 0 | 0 | 0 |
| Bird 123 | Site 3 | ANHU | AHY | M | 3D6.1D593D460E | 2/26/2018 | 3/8/2018 | 3/26/2018 | 10 | 28 | 4 | 0 | 0.0 | 0 | 0 | 0 |
| Bird 124 | Site 3 | ALHU | UNK | F | 3D6.1D593D460F | 11/29/2017 | 12/30/2017 | 12/30/2017 | 31 | 31 | 1 | 0 | 0.0 | 0 | 0 | 0 |
| Bird 125 | Site 3 | ALHU | AHY | M | 3D6.1D593D4610 | 2/25/2018 | 3/25/2018 | 3/25/2018 | 28 | 28 | 1 | 0 | 0.0 | 0 | 0 | 0 |
| Bird 126 | Site 3 | ALHU | UNK | M | 3D6.1D593D4611 | 11/29/2017 | 1/16/2018 | 3/31/2018 | 48 | 122 | 33 | 292 | 0.4 | 0 | 0 | 22 |
| Bird 127 | Site 3 | ALHU | AHY | M | 3D6.1D593D4612 | 2/26/2018 | 3/7/2018 | 3/8/2018 | 9 | 10 | 2 | 0 | 0.0 | 0 | 0 | 0 |
| Bird 128 | Site 3 | ANHU | AHY | F | 3D6.1D593D4614 | 2/25/2018 | 3/25/2018 | 3/25/2018 | 28 | 28 | 1 | 0 | 0.0 | 0 | 0 | 0 |
| Bird 129 | Site 3 | ANHU | AHY | M | 3D6.1D593D7827 | 2/26/2018 | 2/26/2018 | 2/28/2018 | 0 | 2 | 2 | 0 | 0.0 | 0 | 0 | 0 |
| Bird 130 | Site 2 | ANHU | AHY | M | 3D6.1D593D782B | 2/1/2018 | 2/28/2018 | 3/31/2018 | 27 | 58 | 31 | 4326 | 9.6 | 10 | 0 | 121 |
| Bird 131 | Site 3 | ALHU | AHY | F | 3D6.1D593D7835 | 2/26/2018 | 3/25/2018 | 3/25/2018 | 27 | 27 | 1 | 0 | 0.0 | 0 | 0 | 0 |
| Bird 132 | Site 3 | ANHU | AHY | F | 3D6.1D593D7838 | 2/26/2018 | 2/26/2018 | 3/25/2018 | 0 | 27 | 7 | 0 | 0.0 | 0 | 0 | 0 |
| Bird 133 | Site 3 | ANHU | AHY | F | 3D6.1D593D7839 | 2/26/2018 | 2/26/2018 | 3/31/2018 | 0 | 33 | 32 | 43 | 0.1 | 0 | 0 | 11 |
| Bird 134 | Site 3 | ANHU | AHY | M | 3D6.1D593D7840 | 2/26/2018 | 2/26/2018 | 3/31/2018 | 0 | 33 | 33 | 195 | 0.1 | 0 | 0 | 32 |
| Bird 135 | Site 3 | ANHU | AHY | M | 3D6.1D593D7843 | 2/26/2018 | 3/2/2018 | 3/31/2018 | 4 | 33 | 24 | 33 | 0.3 | 0 | 0 | 11 |
| Bird 136 | Site 3 | ANHU | AHY | M | 3D6.1D593D7845 | 2/26/2018 | 3/2/2018 | 3/7/2018 | 4 | 9 | 2 | 0 | 0.0 | 0 | 0 | 0 |
| Bird 137 | Site 2 | ANHU | AHY | M | 3D6.1D593D7848 | 2/1/2018 | 2/1/2018 | 2/28/2018 | 0 | 27 | 27 | 17857 | 10.0 | 0 | 0 | 615 |
| Bird 138 | Site 3 | ALHU | AHY | M | 3D6.1D593D786B | 2/26/2018 | 3/25/2018 | 3/25/2018 | 27 | 27 | 1 | 0 | 0.0 | 0 | 0 | 0 |
| Bird 139 | Site 3 | ALHU | AHY | M | 3D6.1D593D787A | 2/26/2018 | 3/6/2018 | 3/25/2018 | 8 | 27 | 5 | 0 | 0.0 | 0 | 0 | 0 |
| Bird 140 | Site 2 | ANHU | AHY | M | 3D6.1D593D787F | 2/1/2018 | 2/1/2018 | 3/31/2018 | 0 | 58 | 38 | 6032 | 13.4 | 11 | 0 | 120 |
| Bird 141 | Site 3 | ANHU | AHY | M | 3D6.1D593D7880 | 2/26/2018 | 2/26/2018 | 3/6/2018 | 0 | 8 | 9 | 31 | 0.5 | 0 | 0 | 21 |
