# Supplementary figures and images for: Use of RFID technology to characterize feeder visitations and contact network of hummingbirds in urban habitats
Source: PLoS One. 2018 Dec 12;13(12):e0208057. doi: 10.1371/journal.pone.0208057 (PMC6291107; doi:10.1371/journal.pone.0208057)

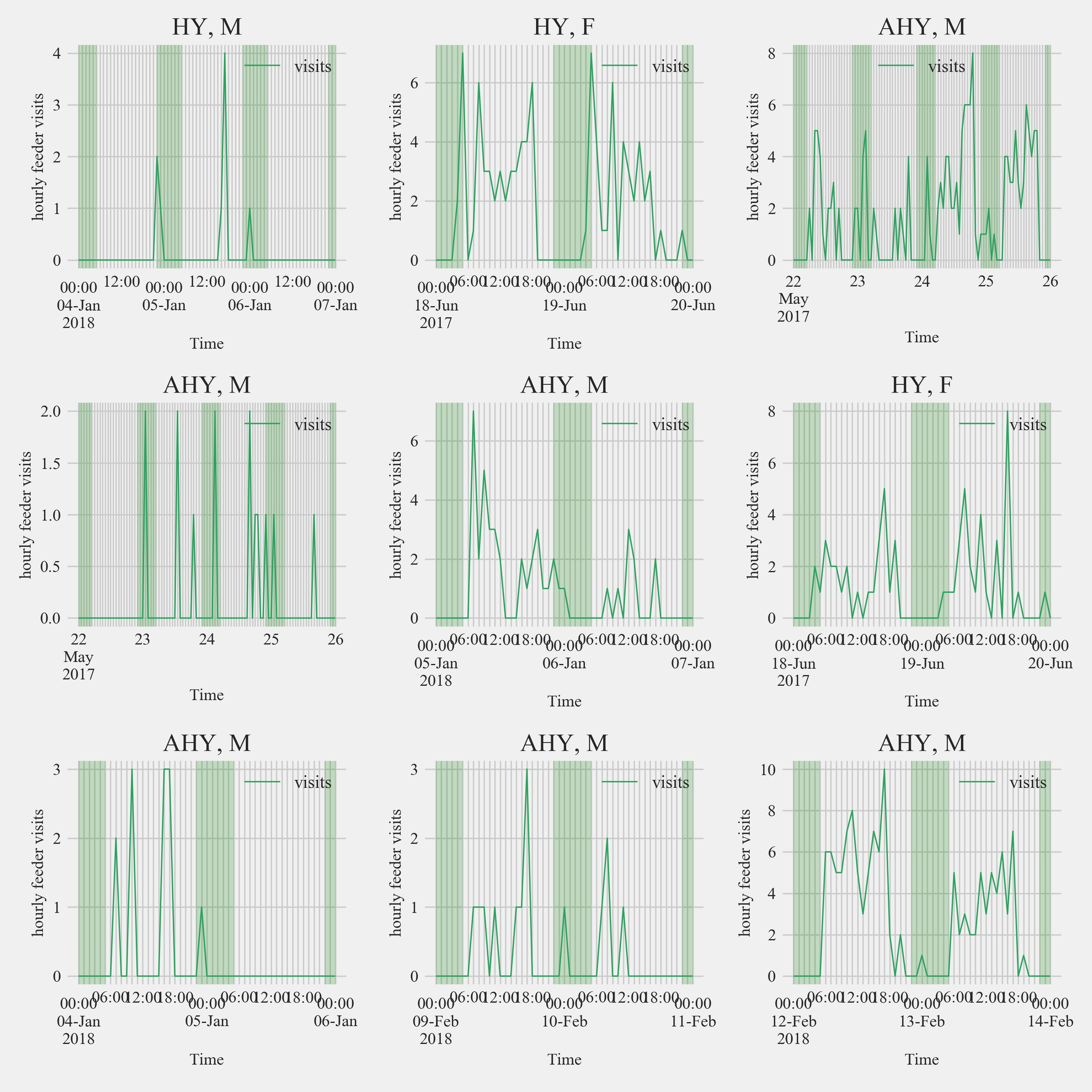

Supplement: S1 Fig — This nocturnal activity was only seen at study site 2 in northern California. The shaded portion represents night time. Days preceding and succeeding the night activity are included showing overall activity around the nocturnal activity. (TIF) [file pone.0208057.s002.tif]

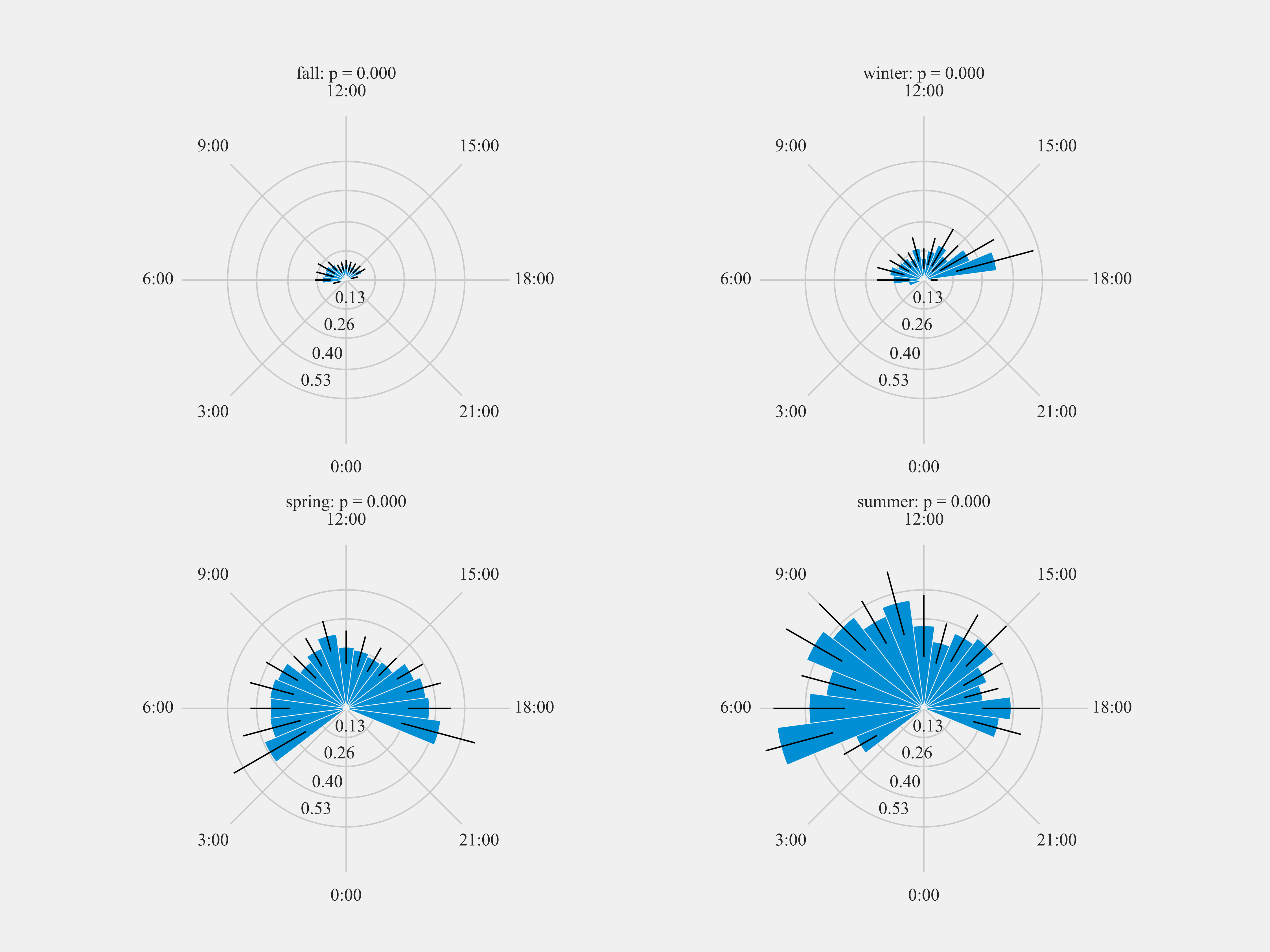

Supplement: S2 Fig — The bar denotes the mean hourly visits by hummingbirds and black lines show the standard error. All distributions were statistically non-uniform (probability values reported by season). (TIF) [file pone.0208057.s003.tif]

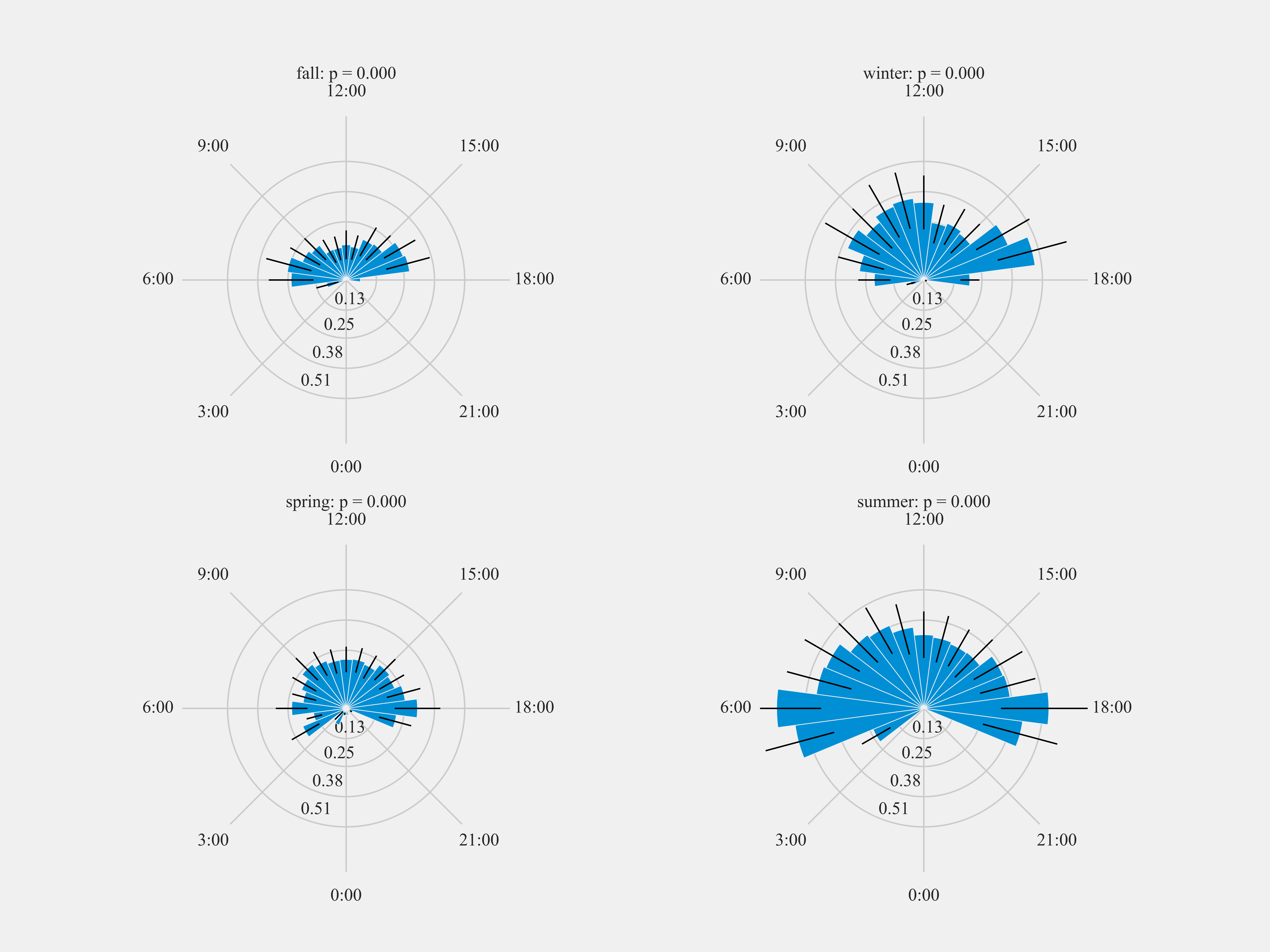

Supplement: S3 Fig — The bar denotes the mean hourly visits by hummingbirds and black lines show the standard error. All distributions were statistically non-uniform (probability values reported by season). (TIF) [file pone.0208057.s004.tif]

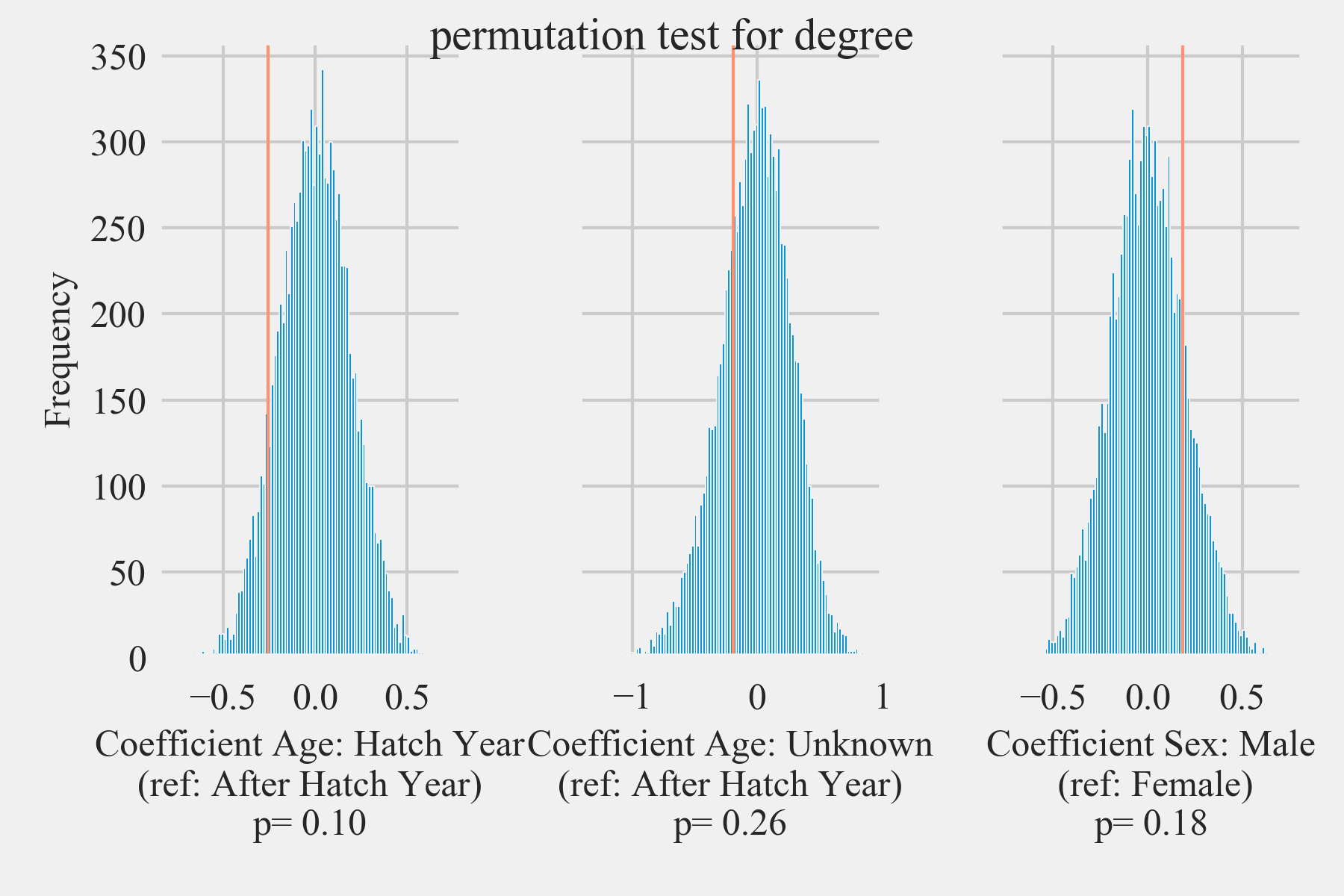

Supplement: S4 Fig — Blue lines show the distribution of coefficients after 10,000 permutations. Red lines show original coefficients. (TIF) [file pone.0208057.s005.tif]

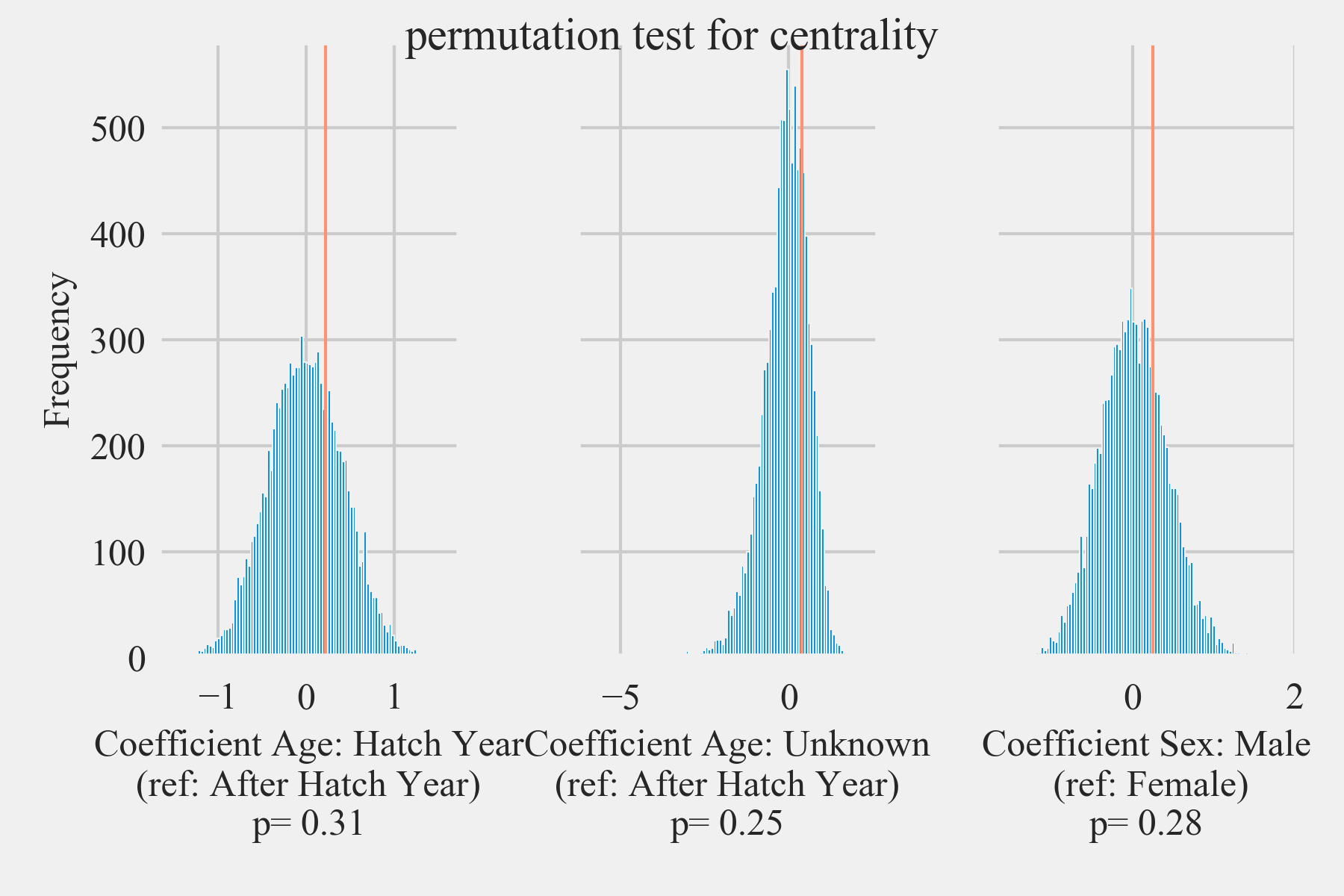

Supplement: S5 Fig — Blue lines show the distribution of coefficients after 10,000 permutations. Red lines show original coefficients. (TIF) [file pone.0208057.s006.tif]
